# A consensus-based framework for conducting and reporting osteoarthritis phenotype research

**DOI:** 10.1186/s13075-020-2143-0

**Published:** 2020-03-20

**Authors:** W. E. van Spil, S. M. A. Bierma-Zeinstra, L. A. Deveza, N. K. Arden, A.-C. Bay-Jensen, V. Byers Kraus, L. Carlesso, R. Christensen, M. Van Der Esch, P. Kent, J. Knoop, C. Ladel, C. B. Little, R. F. Loeser, E. Losina, K. Mills, A. Mobasheri, A. E. Nelson, T. Neogi, G. M. Peat, A.-C. Rat, M. Steultjens, M. J. Thomas, A. M. Valdes, D. J. Hunter

**Affiliations:** 1 Department of Rheumatology & Clinical Immunology, University Medical Center Utrecht, PO Box 85500, 3508 GA Utrecht, The Netherlands; 2 Department of General Practice, Erasmus MC University Medical Center Rotterdam, Rotterdam, The Netherlands; 3 Department of Orthopedic Surgery, Erasmus MC University Medical Center Rotterdam, Rotterdam, The Netherlands; 4 Department of Rheumatology, Royal North Shore Hospital and Institute of Bone and Joint Research, Kolling Institute, University of Sydney, Sydney, Australia; 5 University of Oxford, Oxford, UK; 6 University of Sydney, Sydney, Australia; 7 Rheumatology, Nordic Bioscience, Herlev, Denmark; 8 School of Medicine, Duke University, Durham, USA; 9 Université de Montréal, Montréal, Canada; 10 Musculoskeletal Statistics Unit, The Parker Institute, Copenhagen University Hospital, Bispebjerg and Frederiksberg, Frederiksberg, Denmark; 11 Research Unit of Rheumatology, Department of Clinical Research, Odense University Hospital, University of Southern Denmark, Odense, Denmark; 12 Reade, Center of Rehabilitation and Rheumatology, Amsterdam, The Netherlands; 13 Curtin University, Bentley, Australia; 14 University of Southern Denmark, Odense, Denmark; 15 Department of Health Sciences, VU Amsterdam, Amsterdam, The Netherlands; 16 Merck KGaA, Darmstadt, Germany; 17 Raymond Purves Labs, Institute of Bone and Joint Research, Kolling Institute, University of Sydney, Sydney, Australia; 18 Thurston Arthritis Research Center, University of North Carolina, Chapel Hill, NC USA; 19 Brigham and Women’s Hospital, Boston, USA; 20 Department of Health Professions, Macquarie University, Sydney, Australia; 21 Research Unit of Medical Imaging, Physics and Technology, Faculty of Medicine, University of Oulu, Oulu, Finland; 22 School of Medicine, Boston University, Boston, USA; 23 Arthritis Research UK Primary Care Centre, Research Institute for Primary Care & Health Sciences, Keele University, Staffordshire, UK; 24 Haywood Academic Rheumatology Centre, Midlands Partnership NHS Foundation Trust, Haywood Hospital, Staffordshire, UK; 25 Université de Lorraine, EA 4360 Nancy, France; 26 School of Health and Life Sciences, Glasgow Caledonian University, Glasgow, UK; 27 School of Medicine, University of Nottingham, Nottingham, UK

**Keywords:** Osteoarthritis, Phenotypes, Framework, Consensus (maximum 10)

## Abstract

**Background:**

The concept of osteoarthritis (OA) heterogeneity is evolving and gaining renewed interest. According to this concept, distinct subtypes of OA need to be defined that will likely require recognition in research design and different approaches to clinical management. Although seemingly plausible, a wide range of views exist on how best to operationalize this concept. The current project aimed to provide consensus-based definitions and recommendations that together create a framework for conducting and reporting OA phenotype research.

**Methods:**

A panel of 25 members with expertise in OA phenotype research was composed. First, panel members participated in an online Delphi exercise to provide a number of basic definitions and statements relating to OA phenotypes and OA phenotype research. Second, panel members provided input on a set of recommendations for reporting on OA phenotype studies.

**Results:**

Four Delphi rounds were required to achieve sufficient agreement on 11 definitions and statements. OA phenotypes were defined as subtypes of OA that share distinct underlying pathobiological and pain mechanisms and their structural and functional consequences. Reporting recommendations pertaining to the study characteristics, study population, data collection, statistical analysis, and appraisal of OA phenotype studies were provided.

**Conclusions:**

This study provides a number of consensus-based definitions and recommendations relating to OA phenotypes. The resulting framework is intended to facilitate research on OA phenotypes and increase combined efforts to develop effective OA phenotype classification. Success in this endeavor will hopefully translate into more effective, differentiated OA management that will benefit a multitude of OA patients.

## Background

There has been longstanding acceptance of the heterogeneity of osteoarthritis (OA), but this topic is attracting increasing interest given an expanding armamentarium for classification (biological, psychosocial, and statistical); new insights into the pathophysiology, prognosis, and patterns of response to new and existing interventions; and a general move towards personalized care to improve efficiency and effectiveness [1]. A specific development more recently has been to invoke the concept of OA phenotypes. According to this concept, OA is composed of a number of phenotypes that may be present to a varying extent among patients spanning the spectrum of disease [2]. These phenotypes may differ in their compatibility with study designs in research and diagnostic and therapeutic strategies in clinical care. Although seemingly plausible, a wide range of views exist on how best to operationalize this concept, and similarly, a variety of approaches have been used to explore heterogeneity in empirical studies. Studies focused on OA phenotypes published up to day differ in approach, criteria to distinguish phenotypes, and their outcomes. Furthermore, different papers used different and sometimes confusing terminologies. A more consistent use of terminology would increase the synergy between studies and facilitate the progress of the field as a whole. Furthermore, to allow effective comparison between studies and meta-analyses, complete reporting of relevant data is important.

In the field of back pain, the pathway from basic research to successful phenotyping has been argued as composing of a number of steps [3]. First, there are studies of assessment methods that could potentially provide important data on one or more phenotypes. For example, one may develop a new imaging technique to assess the glycosaminoglycan content of articular cartilage. Second, hypothesis-generating studies aim to determine which characteristics identify people in clinically important subgroups. For example, a biochemical marker level could be higher in a particular subgroup of knee OA patients. Third, hypothesis-testing studies test a priori hypotheses about subgrouping effects in samples of people independent from, but similar to, those people involved in the hypothesis-setting phase. Studies in this phase typically follow a more stringent approach than those at the hypothesis-generating stage. For example, the biochemical marker is now evaluated in a larger, well-characterized patient sample and might include healthy controls. Fourth, relatively narrow validation studies attempt to replicate findings of hypothesis-testing studies in independent samples of people who are similar to those originally studied. Fifth, broader validation studies try to replicate the findings of hypothesis-generating studies in independent samples from broader populations than those originally tested. For example, the biochemical markers for phenotyping OA patients in specialist outpatient clinics would be tested in a primary care setting. Sixth and last, impact-analysis studies examine the capacity of a specific phenotyping method in routine care settings to change clinical decision-making, improve patient outcomes, and/or increase health system efficiency.

The current project aimed to provide a widely supported framework for designing and conducting research along the pathway from basic research to successful clinical application of OA phenotyping, through consensus on a number of definitions and conceptual statements and a set of reporting recommendations. Ultimately, if adopted widely, this should contribute to a more coordinated research effort.

## Methods

The framework consisted of two main parts. First, a panel of researchers with expertise in OA phenotype research commented on a number of basic definitions and conceptual statements in an online Delphi exercise. Second, the panel provided input on reporting recommendations in a face-to-face meeting.

### Panel composition

The panel consisted of 25 members. Panel members were selected to encompass an array of expertise in OA related topics, career stages, and geographical origins. Each of the members had demonstrable experience in phenotype research, as became evident from publications in peer-reviewed scientific journals. The panel was composed and led by a core group (WEvS, SBZ, LAD, DJH).

### Basic definitions and statements

We used the Delphi Decision Aid hosted on the ForecastingPrinciples.com website that was originally developed by J. Scott Armstrong (University of Pennsylvania). It is managed by Kesten C. Green (University of South Australia), and the software is maintained by Saint Louis Integration (stlouisintegration.com).

An initial set of statements was developed by the core group. Statements were based on points that became apparent from literature data on OA phenotypes [1, 4] and phenotype studies in other diseases [3, 5]. All panel members were then invited to score every statement on a range from 0 to 100% (0% meaning no agreement and 100% meaning complete agreement) and provide comments to explain and contextualize the scores. They could also suggest additional statements. For each subsequent round, statements were adapted by the core group in response to scores and comments. In this process, some statements were combined and others split up. Rounds were continued until all statements scored ≥ 80% on average.

Panel members were provided with a document showing anonymized scores and comments from previous Delphi rounds (Additional file 1). The document also explained how and why statements were adapted between rounds. For any particular round, panel members were not aware of scores and comments from other panel members during the time the round was open.

### Reporting recommendations

Based on similar initiatives for research publications in general, or in other fields, a set of reporting recommendations was compiled by the core group [6–8]. These recommendations were discussed in a meeting with panel members on 29 April 2018 and adapted using the synthesis of these discussions. Members who could not attend the meeting were given the opportunity to provide input via email.

## Results

### Basic definitions and statements

**Fig. 1:**
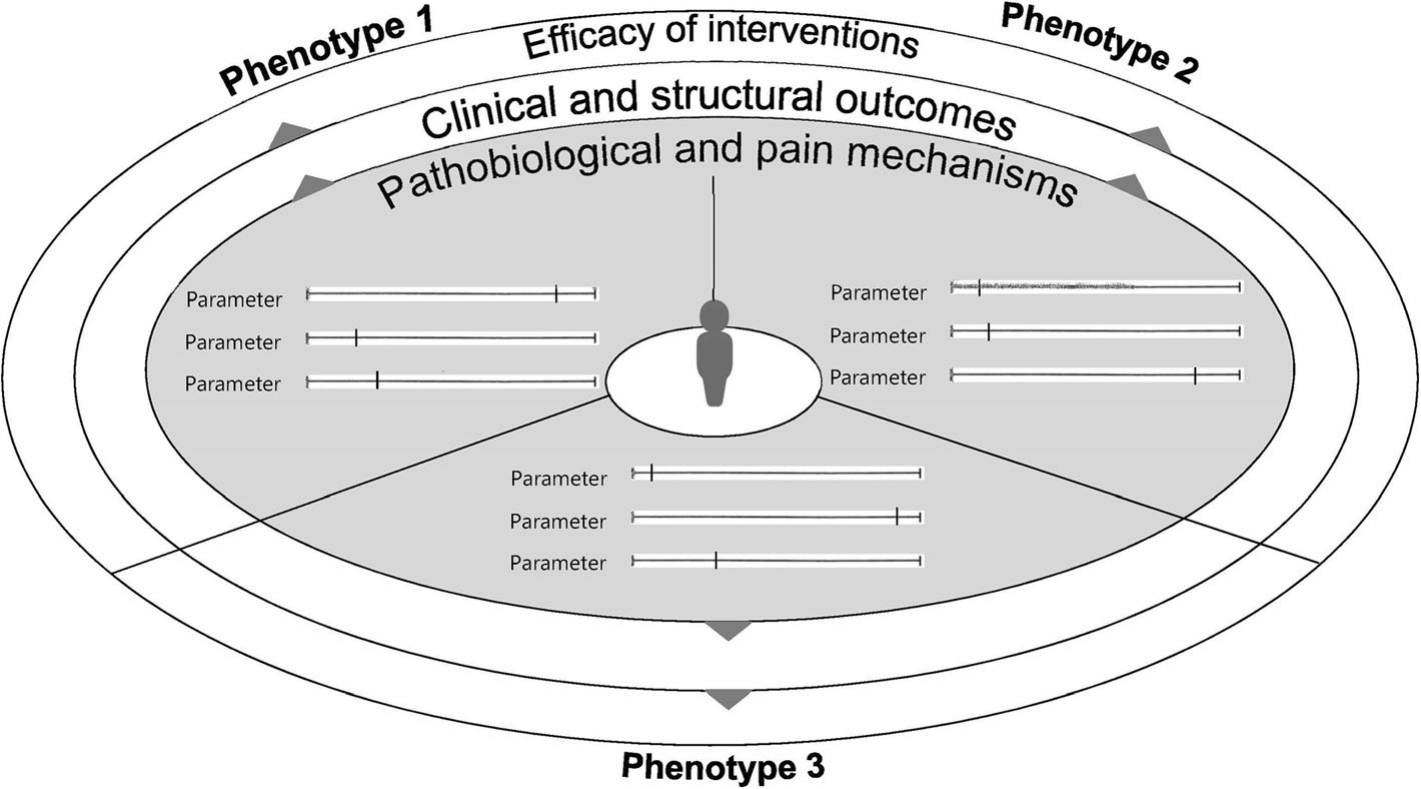
Schematic overview of the general concept behind a number of the statements from the Delphi exercise

Four Delphi rounds were required to achieve sufficient agreement on 11 statements (see Tables 1 and 2). OA phenotypes were defined as subtypes of OA that share distinct underlying pathobiological and pain mechanisms and their structural and functional consequences. This and some of the other concepts in the statements are also summarized in Fig. 1.

**Table 1:** Overview of the Delphi rounds

Round 1	16 statements	16 January to 25 February 2018	22 respondents
Round 2	12 statements	15 February to 12 March 2018	21 respondents
Round 3	11 statements	21 March to 21 April 2018	21 respondents
Round 4	6 statements	13 June to 5 July 2018	20 respondents

Overview of the four Delphi rounds that were run in total. For every round, it is shown how many statements were open for scoring in that round, the period it was open, and how many of the total of 25 panel members responded

**Table 2:** Final statements on OA phenotypes

Number	Statement	Mean score	Distribution of scores (minimum—25% percentile—75% percentile—maximum)
1	OA phenotypes are subtypes of OA that share distinct underlying pathobiological and pain mechanisms and their structural and functional consequences.	86	60—80—94—100
2	OA phenotypes can become apparent in differences in risk factors, prognostic factors, nature and extent of symptoms and signs, disease trajectory, and/or responsiveness to particular treatments or treatment in general.	89	70—80—99—100
3	An OA phenotype classification system is likely to consist of input variables that together reflect (the likelihood of) the presence of one or more pathobiological and pain mechanisms.	88	70—80—94—100
4	Classification systems are likely to use one or more measures from either one or more domains (e.g., imaging markers, biochemical markers, and pain) to identify a clinically relevant OA phenotype or phenotypes.	86	60—80—95—100
5	The potentially identified phenotype(s) should differ from others in terms of clinically relevant disease-driving factors and/or outcomes.	87	50—80—99—100
6	Research efforts may initially lead to multiple proposed phenotype classification systems. Eventually, these should be aligned and come together in one.	84	65—72—94—100
7a	Differences in the disease stage may cause different results from OA phenotyping studies between study populations. It is likely that the nature and course of disease stages may differ between patients and phenotypes.	82	50—80—90—100
7b	Disease stage(s) of the study population should always be reported. Reasons to take or not take disease stage into account in the analyses (e.g., to adjust for confounding or look for interaction) should be weighted for every study.	84	50—80—99—100
8a	Some components of pathobiological and pain mechanisms in OA may be similar between different joints such as knee and hip (e.g., synovitis, central pain perception), while others may differ (e.g., menisci, femoral head shape). The decision to extrapolate findings from one joint to another, or not, should be justified.	86	50—80—94—100
8b	Phenotype classification systems can be designed for individual joints or systemically (e.g., for multiple joints in one patient), depending on the pathobiological and pain mechanism that is under study and the goal of the study.	86	70—80—90—100
9	Data-driven approaches for constructing phenotype classification systems are generally preferable over expert opinion-based approaches, as long as they are performed using high-quality data and appropriate statistics, are reproducible, and have clinical validity, relevance, and applicability as judged by experts in the field.	91	70—86—100—100

Overview of the final statements that resulted from the Delphi exercise. The level of agreement among panel members is indicated for every statement by the mean score (0% meaning no agreement and 100% meaning complete agreement) and the distribution of individual scores

Every OA phenotype encompasses a number of typical pain and/or pathobiological mechanisms. People with OA can be assessed for the presence of one or more parameters that reflect these mechanisms. Every person can have characteristics of one or more phenotypes. Every OA phenotype relates to characteristic clinical and structural outcomes and, with that, to the effectiveness of particular interventions.

### Reporting recommendations

Twelve panel members attended the face-to-face meeting. All panel members were given the opportunity to provide input via email. Reporting recommendations that followed from the panel meeting are summarized in Table 3 and discussed below. These recommendations are anticipated to be useful for authors, reviewers, and editors in the process of writing, reviewing, and publishing reports. Importantly, these criteria are not to be used as quality markers, as at this point, there are insufficient data to support any such decisions. Likewise, it was decided that there were not enough data to support the weighting of individual items. Finally, it is important to emphasize that these recommendations are in no way intended to restrict researchers in their approach to identifying OA phenotypes or determine overlaps between them.

**Table 3:** Reporting recommendations

**General study characteristics**	
Availability of a prespecified research protocol	
Study design: observational cohort, case-control, clinical trial, animal study, other	
Primary goal and setup of the original study, when the phenotype approach is not the primary goal of the study. Cite references/registrations when available	
Intended goal(s) and context(s) of the pursued phenotype classification (e.g., to have prognostic or therapeutic consequences)	
Position of the study with respect to its stage in phenotyping (i.e., study of assessment method, hypothesis-setting, hypothesis-testing, narrow validation, broad validation or impact analysis)	
**Study population**	
Setting: general population, general practitioner, rheumatological and/or orthopedic practice, etc.	
Flow diagram of participants selection process/sampling	
Sample size, dropouts	
Demographics	
Clinical OA characteristics (e.g., pain, function)	
Structural OA characteristics (e.g., radiographic parameters)	
**Data collection**	
Variable(s) for the assumed pathobiological and/or pain mechanisms under study • Explanation of how and why the variable(s) is (are) anticipated to reflect the mechanism(s) • Statement of the quality of the variable(s), when available	
Follow-up time points for each of the variables (longitudinal studies)	
Outcome parameter(s) (i.e., structural and functional consequences of the phenotype(s))	
**Statistical analysis**	
Availability of a prespecified statistical analysis plan	
Analytical approach (supervised, unsupervised*) and rationale	
Power, sample size considerations	
Methods to adjust for potential confounders/effect modifiers and to handle missing data	
Criteria for the distinction between phenotypes and whether these were predefined	
Criteria for clinical relevance and/or applicability and whether these were predefined	
Any sensitivity analyses	
Methods to determine reproducibility/consistency	
Availability of datasets and syntaxes to other investigators (e.g., website, contact details)	
**Appraisal**	
Underlying pathobiological and/or pain mechanisms	
Potential clinical relevance and applicability	
Internal validity, potential sources of bias	
External validity, generalizability	
Comparison with other phenotype classifications/literature data, when possible	
Relevance and consequences of the present work for future research	
Financial/commercial interests, funding sources	

*Supervised statistical methods require output variables to be available and serve to estimate functions that best approximate the relationship between the input and output variables in the dataset (e.g., linear regression). Unsupervised statistical methods are not provided with output variables but are concerned with uncovering structures within datasets without prior knowledge of how the data are organized (e.g., principal component analysis)

### General study characteristics

Currently, most datasets used for OA phenotype research are datasets from other studies or trials that are then secondarily used for phenotyping. Knowledge about the setting and characteristics of the original study are important for proper interpretation of the outcomes of the subsequent phenotype analysis. The original study goals and design will determine the contents of the dataset and the potential outcomes of the phenotype analyses. For example, opportunities for phenotype analysis in a dataset that is originated from a clinical trial will be different from analyses performed with data from an observational study. In keeping with this concept, some researchers may investigate phenotypes that differ in response to treatment, while others may explore phenotypes that differ in natural disease course.

### Study population

The characteristics of the study population are important to take into account when interpreting the validity of the results of the OA phenotype analysis and for comparing results between studies. For example, the potential to identify particular phenotypes might be different between populations with and without OA pain or between patients from general practice and orthopedic clinics. Likewise, non-random subject selection or dropout in observational or interventional studies can affect the study results. For example, a phenotype that is non-responsive to a particular treatment in a clinical trial might show higher dropout rates and thereby provide less follow-up data for subsequent phenotype analysis.

### Data collection

It is likely that every phenotype will be characterized by one or more parameter prognostic factors relating to one or more pathobiological or pain mechanisms that are critical for that particular phenotype. Usually, there are multiple ways to assess and/or monitor OA and these may differ importantly in their ability to reflect the pathogenetic and/or pain mechanisms of interest and in their technical characteristics (e.g., accuracy, precision, reproducibility). Therefore, it is considered important that strengths and weaknesses of the available set of parameters for the phenotype analysis are understood and discussed. For example, subchondral bone structure can be assessed by different imaging techniques and these may or may not be supplemented with biochemical markers of bone metabolism. Biochemical markers might, however, be less specific for the joint tissue of interest or be more subject to noise. The available follow-up time and number of time points may also affect the ability of parameters to differentiate between potential phenotypes. For example, biochemical markers compared with imaging markers might be more dynamic and require shorter follow-up times to show differences in disease course or treatment response between phenotypes.

### Statistical analysis

The panel members considered data-driven approaches valuable for gaining insights into OA phenotypes that extend beyond current knowledge. However, data-driven approaches are often rather sensitive to changes in the particular features of the analysis (e.g., a clustering method, number of subgroups, methods to handle missing data) and selection bias. The features may be fine-tuned in an iterative process whereby outcomes are compared back and forth between settings. This process may be more or less subjective and/or be performed according to prespecified criteria. Irrespective of the approach, the clinical relevance of the potentially identified phenotypes should be accounted for in the analysis plan. For example, differences in pain course between phenotypes should be clinically meaningful. Sensitivity analyses (e.g., repeated analysis with different cutoffs) and methods to describe consistency and reproducibility (e.g., internal or external validity) are considered particularly important for data-driven techniques. Access to the dataset(s) and syntax(es) for other investigators to repeat and/or extend analyses is encouraged.

### Appraisal

For an identified OA subgroup to be considered a distinct phenotype, the extent to which its main underlying pathobiological or pain mechanism(s) can be assumed to differ from others should be made clear. Explaining similarities and differences in relation to the existing literature may highlight consistency of findings across studies and/or point out how observable differences might have occurred. It is also advised to discuss how the identified phenotype(s) might impact future research and practice (e.g., external validity, potential therapeutic consequences).

## Discussion

This OA phenotype framework is intended to facilitate research on OA phenotypes and increase combined efforts to attain effective OA phenotype classification, through providing a number of coherent definitions and statements and a set of reporting recommendations that were supported by a panel of researchers with relevant expertise. The provided framework got focused around distinct pathobiological and/or pain mechanisms. This focus is in line with the ultimate goal to develop phenotype-specific interventions, targeted at these distinct mechanisms. A number of studies argue in favor of the actual existence of subgroups with distinct pathobiological and/or pain mechanisms [9–12]. Further success in this endeavor depends on the adoption of the currently proposed framework in the field. This will hopefully translate into more effective, differentiated OA management that will benefit a multitude of OA patients. Although we aimed to codify a representative set of shared opinions of individuals working in the field of OA phenotypes, we realize that insights will no doubt evolve over time and that updating the framework will likely be required in the future as the field matures and more data will become available. The ultimate success of such an initiative will require consistent and wide implementation.

## Conclusions

A wide range of views exist on how best to operationalize the concept of OA phenotypes. The current initiative provides consensus-based definitions, statements, and reporting recommendations to the OA phenotype research field, supported by an international panel of researchers with relevant expertise. Implementation of these is considered important to standardize and synergize the wide range of research activities that are currently being deployed in this multidisciplinary field. Success in this endeavor will hopefully translate into the consistent identification of distinct phenotypes and more effective, differentiated OA management.

## Supplementary information

**Additional file 1: Appendix 1.** Overview of the statements and panel scores for every Delphi round.

## Data Availability

Requests for data and materials relating to this publication can be submitted to the corresponding author.

## Abbreviation

OA: Osteoarthritis
