# Supplementary material for: A consensus-based framework for conducting and reporting osteoarthritis phenotype research
Source: Arthritis Res Ther. 2020 Mar 20;22:54. doi: 10.1186/s13075-020-2143-0 (PMC7083005; doi:10.1186/s13075-020-2143-0)
Supplement: Supplementary file 1 — Additional file 1: Appendix 1. Overview of the statements and panel scores for every Delphi round. [file 13075_2020_2143_MOESM1_ESM.docx]

**Transition round 1 to 2**

**Former statements:**

- Patient subgroups with different longitudinal trajectories of an OA-related variable (e.g., pain, joint space width) represent OA phenotypes (*score:* mean 59, SD 27, median 60).

- Patient subgroups with different predominating OA-related variables (e.g., synovitis vs. osteophytes) represent OA phenotypes (*score:* mean 72, SD 17, median 70).

- Patient subgroups differing with regard to response to a particular treatment represent different OA phenotypes (*score*: mean 69, SD 23, median 60).

- Patient subgroups differing with regard to prognostic factors represent OA phenotypes (*score:* mean 62, SD 26, median 60).

- Patient subgroups differing with regard to etiological backgrounds represent OA phenotypes (*score:* mean 66, SD 25, median 70).

- OA subtype/subgroup is synonymous with OA phenotype (*score:* mean 63, SD 30, median 60).

- *Many experts think that trajectories, predominating OA features, treatment responses, and prognostic risk factors each capture different aspects of the concept of phenotypes, but that they are not synonymous.*
- *Multiple experts think subgroups are not similar to subtypes. Most think subtyping comes closer to phenotyping, because it implies shared underlying disease mechanisms within a subtype, as opposed to subgroups that can be defined by any criterion or even by chance.*

**New statement 1:** OA phenotypes are subtypes of OA that share, or can reasonably be assumed to share, distinct underlying functional or pathobiological mechanisms.

**New statement 2:** OA phenotypes can become apparent in differences in risk factors, nature and extent of symptoms and signs, disease trajectory, and/or responsiveness to particular treatments or treatment in general.

**Former statements:**

- Overlap between phenotypes is to be expected (*score:* mean 78, SD 19, median 80).
- Patient subgroups should preferably be defined using parameters from different OA domains (e.g., structural, functional, psychological) (*score:* mean 70, SD 22, median 70).
- Not all domains are equally efficient in separating OA phenotypes (e.g., pain vs. radiographic joint space narrowing) (*score:* mean 80, SD 17, median 80).
- The relevance of OA phenotypes defined from different domains is different (e.g., pain vs. radiographic joint space narrowing) (*score:* mean 67, SD 23, median 70).
- *Multiple experts express a preference of clinically relevant and/or modifiable OA-related variables to be used in phenotype classification (e.g. synovitis and pain over osteophytes and K&L grade).*
- *Multiple experts do not fully understand or do object to the terms “relevance” and “efficiency” of OA-related variables and domains. These terms, after all, depend on the context of the proposed phenotype classification.*
- *Experts do acknowledge the complexity of OA, but not all agree that phenotypes should be classified from OA-related variables from different domains. Multiple experts also emphasize that such an approach would have to be very comprehensive and be very costly. They also doubt whether it would be feasible at the moment, considering limitations in our ability to fully capture every domain appropriately.*

**New statement 3:** A system to classify OA patients for one or more phenotypes should consist of input variables that together reflect (the likelihood of) the presence of one or more functional or pathobiological mechanisms in these patients.

**New statement 4:** To ascertain that OA phenotype classification systems could eventually influence decision-making in clinical trials and practice, the potentially identified phenotype(s) should differ from others regarding disease-driving factors and/or outcomes, e.g. disease trajectory and/or responsiveness to particular treatments or treatment in general, etc.

**New statement 5:** As OA is a complex, multidimensional disease, it is likely that for classification systems to be effective, input variables should together assess OA from different perspectives (e.g. physical examination, imaging, biochemical markers, etc.).

**New statement 6:** Phenotype classification systems can be developed in different contexts and with different goals. Therefore, there could be multiple OA phenotype classification systems that would be used either separately or in combination, e.g., systems that relate to prognosis and systems that relate to treatment response.

**Former statements:**

- Structural OA stages (e.g., Kellgren and Lawrence grade 1 vs. 3) represent different phenotypes (*score:* mean 35, SD 25, median 30)
- Clinical OA stages (e.g., recent onset vs. long-standing pain) represent different phenotypes (*score:* mean 50, SD 30, median 50).
- OA populations for investigating phenotypes should consist of people in a similar OA stage (either clinical or structural) or analyses should at least be adjusted for that (*score:* mean 60, SD 23, median 65).
- *Most experts agree that disease stages are not similar to phenotypes.*
- *Many experts think disease stage is relevant for phenotype classification, but they differ in their argumentation.*

**New statement 7:** Disease stage is likely to be relevant for OA phenotyping, as disease stages in themselves might already differ in the predominance of etiological pathways and our ability to capture their typical characteristics (e.g. minimal vs. extensive cartilage changes, preclinical metabolic derangements vs. overt structural disease, etc.).

**New statement 8:** It should, however, also be acknowledged that the nature and course of disease stages may differ between patients and phenotypes and that our methods for disease staging have their limitations. Reasons to adjust, or not adjust analyses for disease stage should be considered for every study.

**Former statement:** A presumed phenotype comprising just a very limited number of subjects is less relevant (*score:* mean 47, SD 31, median 40).

- *Most experts, some conditionally, acknowledge that a phenotype comprising just a limited number of subjects can be important.*

**New statement 9:** A phenotype classification category comprising just a very limited part of the population as a whole can well be informative, particularly when it is consistently and clearly distinguishable from the others and when it impacts decision making in clinical trials or practice.

**Former statement:** OA in different joints represents different phenotypes (*score:* mean 70, SD 17, median 70).

- *Most experts agree that functional or pathobiological mechanisms may to a certain extent be similar between joints, but that joint-specific mechanisms will play an important role too, e.g. menisci in the knee and femoral head shape in the hip. Moreover, some mechanisms may affect single joints, e.g., trauma, while others will affect multiple joints, e.g. metabolic factors.*
- *Multiple experts mention that phenotype classification systems can be set up either per joint type or systemically, for multiple joints in one patient, depending on the context.*

**New statement 10:** Phenotype classification systems for individual joints can probably not be translated directly to other joints. Although different joints may share common functional or pathobiological OA mechanisms, their consequences probably differ between joints and joint-specific factors (e.g. menisci, femoral head shape) also play a role.

**New statement 11:** Phenotype classification systems can be set up either per joint type or systemically, for multiple joints in one patient, depending on their intended context and goal.

**Former statement:** OA phenotypes derived from data-driven approaches should be valued more highly than those based on expert opinion (*score:* mean 75, SD 19, median 80).

- *Most experts tend to agree with the statement, but multiple experts emphasize the need for high-quality data and statistics for such approaches and the importance for clinical validity, relevance and applicability.*

**New statement 12:** Data-driven approaches for constructing phenotype classification systems are generally preferable over expert opinion-based approaches, as long as they are performed using high-quality data and statistics and with attention to clinical validity, relevance and applicability.

**Detailed results Delphi round 1**

| **Question 1** | OA subtype/subgroup is *synonymous* with OA phenotype. |
| --- | --- |
| **Mean** | 63 |
| **SD** | 30 |
| **Median** | 60 |

Expert's answer: 60

Expert's comment: In the strict sense of the word "phenotype" is just "a set of observable characteristics of an individual". So phenotype MAY be synonomous with some subtypes/subgroups of OA IF they are based on OBSERVABLE characteristics, as this is likely how one divides into sub-types. BUT there will be sub-types/sub-groups that do not have observable characteristics, but are still separate classifications that could be important e.g. you may not be able to OBSERVE if a particular chronic OA joint developed as a result of joint injury, but the history will help you. I wonder if the very concept of using phenotype is not good - rather we shiudl perhaps use the term "endotype" which is "a subtype of a condition, which is defined by a distinct functional or pathobiological mechanism" and thus is likely to have different treatment approaches/targets. This is more clinically relevant that phenotypes/sub-types/sub-groups that have the same pathobiology and trajectory and so should be treated the same....

Expert's answer: 20

Expert's comment: Thee is a Collection Bryde en phenotypes, but a phenotype is defined by disease characteristics. Subgroups, which is different from subtypes, is a term which is already in use drug development and part of the regalatory vocabulary.a subgroup can be defined by a phenotype.

Expert's answer: 90

Expert's comment: I answer this way from the clinical perspective, rather than from an anatomical/histological perspective.

Expert's answer: 90

Expert's comment: I would like phenotype, subtype and subgroup to mean the same thing.

Expert's answer: 30

Expert's comment: I believe phenotype is a concept that involves etiology/disease mechanisms and everything that manifests as a result of this (clinical presentation, laboratory or structural changes). Subgroup, in turn, can be related to any characteristic and does not necessarily represent a phenotype (e.g. people with higher or lower pain levels - may be two different subgroups but not necessarily phenotypes).

Expert's answer: 40

Expert's comment: I think a subtype or subgroup could separate individuals with OA based o clinical signs/symptoms only. Phenotype implies that there is some biological separation as well.

Expert's answer: 30

Expert's comment: OA phenotype suggests a more thorough entity than subtype/subgroup, as phenotype also encompasses assumptions about underlying aetiology and meaning as opposed to subtype/subgroup.

Expert's answer: 20

Expert's comment: Subgroup feels broader - could encompass for example comorbidity pattern, i.e. not a manifestation/expression of OA as such, but may nevertheless be useful for stratifying care

Expert's answer: 80

Expert's comment: subtype or phenotype are defined by common characteristics (actually subgroups could be defined by lottery)

Expert's answer: 0

Expert's comment: No because there may be subgroups within a phenotype

Expert's answer: 80

Expert's comment: I think we need to be more specific ie predictive phenotype = predictive sub-group but not prognostic subgroup

Expert's answer: 70

Expert's comment: It is not exactly the same, but we look at subgroups.

Expert's answer: 20

Expert's comment: By the way: a subtype (says something about the OA type) and subgroup (can be everything) is not the same; - A subtype could be the by hip dysplasia induced hip OA at young age - A subgroup could be women, the very old, etc

| **Question 2** | Patient subgroups with different *longitudinal trajectories* of an OA-related variable (e.g., pain, joint space width) represent OA phenotypes. |
| --- | --- |
| **Mean** | 59 |
| **SD** | 27 |
| **Median** | 60 |

Expert's answer: 90

Expert's comment: The disease outcome/trajectory is important clinically relevant division of patients/phenotypes

Expert's answer: 30

Expert's comment: We should be very clear on the use of vocabulary. Subgroup is a word used in clinical drug development and should be reserved for this. If it stated subtypes my answer would be different

Expert's answer: 70

Expert's comment: I think if these as being potential components or characteristics of phenotypes, rather than phenotypes on their own.

Expert's answer: 25

Expert's comment: I would not like it if phenotypes were outcome based, e.g. through the trajectory of important symptoms like pain and joint damage. My preference is for phenotype definitions based on presumed / evidenced underlying disease mechanisms.

Expert's answer: 90

Expert's comment: This is one sort of phenotype but not the only kind.

Expert's answer: 20

Expert's comment: Based on my previous comment,subgroups with different longitudinal trajectories based on clinical symptoms would represent different subgroups.

Expert's answer: 40

Expert's comment: Not necessarily. Trajectories may be just a spectrum of the different rates of progression influenced by diverse risk factors. I do not believe that this constitute a phenotype although it is important to identify these people with worse prognosis for treatment. However, if a group of patients identified as belonging to a particular trajectory of progression has very defined baseline characteristics, this may help to characterize them as a phenotype.

Expert's answer: 50

Expert's comment: Trajectories can reflect phenotypes, but, again, phenotypes relate to assumptions on an underlying process more than trajectories do.

Expert's answer: 20

Expert's comment: Longitudinal trajectories must also reflect the (lack of) effectiveness of intervention and so this feels more like a way of prognostic way of subgrouping rather than phenotyping

Expert's answer: 90

Expert's comment: a longitudinal trajectory is a characteristic... the objective to phenotype OA patients according to their evolution is an epidemiological objective: to define pronostic factors of different evolutions. the objective of defining phenotype according to for example x raus characteristics is more physiopathologic and explanatory

Expert's answer: 50

Expert's comment: This response is linked with my response to q14. When looking longitudinally we may be assessing people across different disease stages and so what takes priority, disease stage or trajectory of a variable? perhaps the two should be considered simultaneously

Expert's answer: 90

Expert's comment: In my mind this represents the current best way to identify distinct and meaningful phenotypes

Expert's answer: 90

Expert's comment: I agree with this 100% for clinical variables e.g. [ain, but am less certain for purely radiographic variables e.g. JSN

Expert's answer: 70

Expert's comment: In phenotyping, the experience in longitudinal trajectories is not very strong. Difficulties are when patients jump over from one phenotype to another over time.

Expert's answer: 70

Expert's comment: Differences in trajectory is a possibility of distinguishing phenotypes, but not a necessary factor

Expert's answer: 40

Expert's comment: In my opinion a disease phenotype is something that you recognize based on disease characteristics. Prognosis is not such a characteristic, but different phenotypes can differ in prognosis.

| **Question 3** | Patient subgroups with different predominating OA-related variables (e.g., synovitis vs. osteophytes) represent OA phenotypes. |
| --- | --- |
| **Mean** | 72 |
| **SD** | 17 |
| **Median** | 70 |

Expert's answer: 50

Expert's comment: Only useful divisions/phenotypes IF the OA-related variable changes clinical outcome and treatment strategy: less likely with osteophytes more likely with synovitis.

Expert's answer: 70

Expert's comment: I think if these as being potential components or characteristics of phenotypes, rather than phenotypes on their own.

Expert's answer: 80

Expert's comment: this is key to phenotype identification - key factros from a disease mechanism that are present in some patients and absent in others, e.g varus malalignment, widespread pain, synovitis

Expert's answer: 90

Expert's comment: Perhaps these are "clinical" phenotypes and those in question 2 are "outcome" phenotypes?

Expert's answer: 90

Expert's comment: This is the difference that I was talking about in question 1, as opposed to subgroups with different clinical features.

Expert's answer: 70

Expert's comment: As long as these OA-related variables relate to different underlying processes, they may indeed reflect OA phenotypes. Ideally, multiple variables together indicate that different processes underly the predominating features in a subject (e.g., not only osteophyes, but also subchondral bone features, bone biomarkers etc.).

Expert's answer: 60

Expert's comment: Kind of. The presence of particularly manifestations of the condition I agree with, but 'OA-related variable' feels too loose (lots of variables are associated with OA) but are they useful in defining the essence of phenotypes?

Expert's answer: 70

Expert's comment: Yes but this is very broad as there are multiple variables that may be considered OA-related (e.g. does it include differences in pain severity?)

Expert's answer: 50

Expert's comment: Feel more strongly that we should stick to domains and their parameters to define phenotypes. If we continue to use individual variables to define phenotypes, the current state of complexity and heterogeneity will persist.

Expert's answer: 50

Expert's comment: Not a priori, to be able to say this, evidence is required to show they are distinct and meaningful; for instance, based on these different feature, is there a different rate of associated cartilage loss, use of pain meds, gait disturbance, sleep disturbance, etc

Expert's answer: 100

Expert's comment: Again, it is important that we don’t rely just ion imaging/structure based variables

Expert's answer: 30

Expert's comment: One can argue about synovitis, but subgrouping patients as an osteophyte type is not clear to me.

Expert's answer: 90

Expert's comment: I quess that a phenotype should have a predominating OA-related variable (clinical, demographic, tissue-related), to be an OA phenotype. These variables can also linked to the response to treatment and prognosis (see question 4 and 5)

Expert's answer: 70

Expert's comment: Yes, the one group with for example a lot synovitis has a different phenotype than the other group that has not.

| **Question 4** | Patient subgroups differing with regard to *response to a particular treatment* represent different OA phenotypes. |
| --- | --- |
| **Mean** | 69 |
| **SD** | 23 |
| **Median** | 60 |

Expert's answer: 100

Expert's comment: This is THE important phenotype/endotype

Expert's answer: 90

Expert's comment: It treatment is designed to modify components on a causal pathway, then subgroups with a clearly different response to a particular treatment would represent different phenotypes.

Expert's answer: 65

Expert's comment: Ultimately, I would hope that we can map the right treatment to each phenotype - in which case this statement would be true. For some grouping factors (e.g. gender, age) I would however be doubtful that differential response is phenotype-based.

Expert's answer: 85

Expert's comment: ?treatment phenotypes? Not sure a phenotype would be defined by this, but perhaps other clinical or outcome phenotypes would be expected to behave in a certain way with regard to a given treatment.

Expert's answer: 50

Expert's comment: It would depend on the target of the treatment

Expert's answer: 70

Expert's comment: Different phenotypes probably require different, targeted, therapies. Differences in treatment response may, therefore, reflect phenotypes, but are not synonymous. As mentioned before, multiple indicators should corroborate the assumption that differences in treatment response come forth from differences between phenotypes.

Expert's answer: 50

Expert's comment: Possibly, akin to 'treatment-resistant X' could be a useful axis for defining phenotypes although my sense is that this may apply to a relatively narrow set of interventions with a very specific and well-understood mechanism of action

Expert's answer: 30

Expert's comment: I don't believe all predictors of treatment response necessarily represent a phenotype, although phenotypes may have different responses to treatment.

Expert's answer: 50

Expert's comment: Not necessarily because a patient may only respond when in an active phase, but in general there may be specific phenotypes, such as high bone turnover, that may predict response to bone-acting agent

Expert's answer: 100

Expert's comment: Possible the most important phenotype

Expert's answer: 20

Expert's comment: In response to a treatment (a specific one) and the relation to a subgroup is not clear to me.

Expert's answer: 100

Expert's comment: In my opinion, the main goal and clinical relevance of distinguish phenotypes is to target the treatment, therefore this implies that subgroups differ in their treatment response and need different treatments

Expert's answer: 40

Expert's comment: It is possible that a certain phenotype responds differently on a certain treatment, but this is not so per definition

| **Question 5** | Patient subgroups differing with regard to *prognostic factors* represent OA phenotypes. |
| --- | --- |
| **Mean** | 62 |
| **SD** | 26 |
| **Median** | 50 |

Expert's answer: 60

Expert's comment: Not sure - ONLY if the prognostic variable are VERY accurate which then means thsi question is really about disease trajectory. If the prognostic variable is not very accurate than is NOT useful to phenotype by this e.g. osteophytes...

Expert's answer: 50

Expert's comment: If we define prognostic factors as predictors of outcome that are unrelated to treatment exposure (are the same regardless or treatment) and predictors of treatment response we define as treatment effect modifiers, then whether 'patient subgroups differing with regard to prognostic factors represent OA phenotypes' depends on whether those prognostic factors are specific to OA or are more generic to a range of musculoskeletal conditions. If they are specific to OA, then I would think they represent OA phenotypes. If not, they are just generic prognostic factors.

Expert's answer: 70

Expert's comment: Yes for the most part, although not if based on gender or age.

Expert's answer: 50

Expert's comment: More complicated and I believe it depends on whether the prognostic factor is modifiable or non-modifiable. Exhibiting a specific prognostic fact not mean that a person's prognostic trajectory cannot be altered.

Expert's answer: 70

Expert's comment: Prognostic factors probably differ between phenotypes. As before, these terms are not synonymous though.

Expert's answer: 20

Expert's comment: I suspect the major prognostic factors (for outcomes like disability, persistent severe pain) will be very similar across all OA phenotypes. Prognostically-defined subgroups may be more important than phenotypes but I would regard them as different

Expert's answer: 20

Expert's comment: Patient subgroups with the same prognostic score might not necessarily belong to a specific phenotype.

Expert's answer: 50

Expert's comment: It depends on how predictive the “prognostic factors” really are. If they are highly valid and qualified, then they might differentiate subgroups.

Expert's answer: 100

Expert's comment: This and above are where we should be focussing

Expert's answer: 50

Expert's comment: To find clusters of prognostic factors is of high importance. However, using prognostic factors for finding differences between subgroups is not logical to me. Has this been done already?

Expert's answer: 100

Expert's comment: Based on my comment at question 4, I would think that this (a subgroup-specific prognosis, with a subgroup-specific treatment) is the main rationale to distinguish phenotypes

Expert's answer: 40

Expert's comment: If the prognostic factors are phenomena of the disease, yes it could be. But this is not per definition.

| **Question 6** | Patient subgroups differing with regard to *etiological backgrounds* represent OA phenotypes. |
| --- | --- |
| **Mean** | 66 |
| **SD** | 25 |
| **Median** | 70 |

Expert's answer: 30

Expert's comment: ONLY if the eitology changes the disease pathobiology and therefore trajectory and treatment

Expert's answer: 50

Expert's comment: If different aetiological backgrounds result in different rates of OA progression, I would think of this aspect as being a potential component of OA subtypes.

Expert's answer: 90

Expert's comment: This is at the heart of phenotyping for me.

Expert's answer: 70

Expert's comment: If that aetiological background influences the structural disease, compartment affected, potential treatment options and disease course then this could be interpreted as a different phenotype. If the aetiological background has no influence on these factors then there is little use in defining the subgroup.

Expert's answer: 90

Expert's comment: For me, this is the basic definition of phenotypes

Expert's answer: 30

Expert's comment: It feels like this should be important but the problem I have is that I believe OA to be a complex disease with multiple causal components, often cumulative over time, and it seems unlikely that any neat separation by any one causal factor would be useful

Expert's answer: 90

Expert's comment: More importantly if this results in different disease manifestation or course.

Expert's answer: 10

Expert's comment: Etiological backgrounds seems more like risk factors than phenotypes

Expert's answer: 80

Expert's comment: They do indeed, but although important for etiologic research, I think that we should focus on 4 and 5

Expert's answer: 60

Expert's comment: I’m not sure whether this is possible, but could be an interesting study.

Expert's answer: 70

Expert's comment: Differences in etiology is a possibility of distinguishing phenotypes, but not a necessary factor

Expert's answer: 30

Expert's comment: A subgroup with different etiological backgrounds is in my opinion a subtype of OA. People with different etiological background can present with the same phenotype.

| **Question 7** | *Overlap* between phenotypes is to be expected. |
| --- | --- |
| **Mean** | 78 |
| **SD** | 19 |
| **Median** | 80 |

Expert's answer: 80

Expert's comment:

In the strict meaning of phenotype (observable characteristic) then yes, BUT if we mean phenotype as in different pathobiology - then no

Expert's answer: 90

Expert's comment: Overlaps between phenotypes is common in other musculoskeletal phenotypes.

Expert's answer: 95

Expert's comment: YES. Disease mechanisms are not mutually exclusive, and we coined patients fitting into more than one phenotype 'complex OA' in the Dell'Isola paper in PLoS One published last week (http://journals.plos.org/plosone/article?id=10.1371/journal.pone.0191045)

Expert's answer: 75

Expert's comment: It would depend on the method used to identify the subgroups. Statistical analysis can be designed so that no overlap is permitted between groups or the amount of overlap is controlled. However, no point estimate of effect is perfect so it is reasonable to assume there would uncertainty on the margins of classification.

Expert's answer: 80

Expert's comment: I think the different processes within a joint are all related. Therefore, I think it wouldn't be realistic to assume that phenotypes would relate to completely independent underlying processes. Phenotypes will differ in the dominating processes though.

Expert's answer: 70

Expert's comment: There are many dimensions of OA (pain, structure, function etc) and phenotypes within each dimension will possibly overlap with phenotypes from another dimension.

Expert's answer: 70

Expert's comment: overlaping will probably also depends of the "type" of phenotype; phenotypes defined by their pronostic factors will overlap, this will be less true for phenotypes defined by a specific risk factor (e;g: post traumatic OA..)

Expert's answer: 95

Expert's comment: particularly if we agree that they are to be constructed from different domains as stated in the next question.

Expert's answer: 90

Expert's comment: yes

Expert's answer: 90

Expert's comment: It is very difficult to find independent subgroup that do not show any overlap. When it is possible that phenotypes are really independent form each other you will not find any overlap: which I can’t imagine.

Expert's answer: 50

Expert's comment: Depends on the descriptions of the phenotypes

| **Question 8** | Patient subgroups should preferably be defined using parameters from *different OA domains* (e.g., structural, functional, psychological). |
| --- | --- |
| **Mean** | 70 |
| **SD** | 22 |
| **Median** | 70 |

Expert's answer: 100

Expert's comment: Anything else would be under-informed and less potentially useful.

Expert's answer: 95

Expert's comment: Most definitely. Successful phenotyping requires a multifactorial inventory.

Expert's answer: 80

Expert's comment: This seems initially correct, but also requires substantially more information and therefore cost. If a phenotype could be defined in a reasonable way with only easily accessible clinical information, that would be much less effort and cost.

Expert's answer: 90

Expert's comment: Clinically, this would have the most utility.

Expert's answer: 90

Expert's comment: In line with my previous answer, I think that potential underlying processes should always be considered in the context of others. This will, however, not always be completely feasible due to financial or technical factors.

Expert's answer: 50

Expert's comment: It depends on the purpose of the classification - a single approach to classification cannot be expected to fulfil all needs

Expert's answer: 70

Expert's comment: Preferably yes but this approach may not be feasible due to the extraordinary complexity of the disease and phenotypes may need to be defined within each domain.

Expert's answer: 40

Expert's comment: What do you mean ? defining subgroups with a complex index combining different domains ?: it depends of the objective of the use of the phenotype: if this is to analyze an health state and/or its evolution, the answer is yes. if this is to personnalize a treatment (e.g. according to a type of pain) the answer is no

Expert's answer: 85

Expert's comment: Complexity of the disease warrants this

Expert's answer: 20

Expert's comment: Each domain may not contribute each subgroup

Expert's answer: 50

Expert's comment: Some will and some won’t. We shouldn’t be too dogmatic

Expert's answer: 70

Expert's comment: I suppose that this is possible, however, a big dataset is needed to be successful.

Expert's answer: 80

Expert's comment: If the relevant factors for prognosis and targeting treatment are from different domains, than yes.

Expert's answer: 30

Expert's comment: I think that phenotypes should be defined per domain, and not a overall phenotypes including all domains.

| **Question 9** | Not all domains are equally *efficient* in separating OA phenotypes (e.g., pain vs. radiographic joint space narrowing). |
| --- | --- |
| **Mean** | 80 |
| **SD** | 17 |
| **Median** | 80 |

Expert's answer: 100

Expert's comment: I'm using phenotype here as in different disease pathobiology and trajectory.

Expert's answer: 90

Expert's comment: This depends on the important question as to what is the intended purpose or application of the phenotypes.

Expert's answer: 60

Expert's comment: Tend to agree - due to the high or low mechanistic importance of certain domains but most definitely also due to low precision of measures in some domains.

Expert's answer: 90

Expert's comment: I would expect some features to make a much larger impact on separating phenotypes than others, although there would need to be considerations of cost effectiveness as well.

Expert's answer: 100

Expert's comment: There is going to be a difference between modifiable and non-modifiable factors

Expert's answer: 80

Expert's comment: I think the extent of noise, specificity, sensitivity etc. differs between OA-related variables. In line with that, I think some are more efficient than others. This is not necessarily different from other OA research areas.

Expert's answer: 50

Expert's comment: I'm not sure what is meant here by efficiency nor whether 'domain' is the right level. Due to their validity, random error, cost of obtaining, stability over time, etc I can imagine some factors being less useful than others

Expert's answer: 80

Expert's comment: It would be helpful to define what domains we're talking about and what we mean by efficient.

Expert's answer: 70

Expert's comment: This also depends of the objective of the research

Expert's answer: 85

Expert's comment: in previous questions pain and joint space narrowing were referred to as OA variables and here they are domains…It would be helpful to clarify if they are considered to be one or the other or both as it may impact my response to certain questions. Is joint space narrowing a domain?

Expert's answer: 80

Expert's comment: Pain is so heterogeneous that it is not very useful. We need more objective means of identifying different types of pain. Even night pain vs pain at rest etc could assist in making this symptom more informative. I favor a much greater use of objective measures and outcomes to optimize their efficiency.

Expert's answer: 70

Expert's comment: I have difficulties with the word “efficient”. What do you mean with equally efficiency? I assume that it is true when variables with a difference in outcome are not having comparable strength in statistical analyses. Pain on a continuous outcome scale related to ROA on a rational scale. So, I think a agree with this statement (but not sure).

Expert's answer: 80

Expert's comment: Some domains have less impact on prognosis and targeting treatment, so yes.

Expert's answer: 30

Expert's comment: This totally depends on your intention with defining a phenotype within domains. Do you want to treat pain, or do you want to give a disease modifying treatment?

| **Question 10** | The *relevance* of OA phenotypes defined from different domains is different (e.g., pain vs. radiographic joint space narrowing). |
| --- | --- |
| **Mean** | 67 |
| **SD** | 23 |
| **Median** | 70 |

Expert's answer: 60

Expert's comment: Unclear what relevance means- is this clinical relevance?

Expert's answer: 60

Expert's comment: This also depends of the intended purpose of the phenotype classification.

Expert's answer: 50

Expert's comment: I'm not entirely sure I understand this statement to be honest. Context is always important, but the challenge will be to identify phenotypes that create the context rather than phenotypes that depend on the context to have meaning.

Expert's answer: 60

Expert's comment: Wouldn't it depend on why the subgroup/phenotype was described and the margin of difference of each domain/characteristic between different groups? For example, if the subgroup was described for prognostic reasons, then all characteristics may be equally relevant, if defined to describe different structural changes, then pain may not be as relevant.

Expert's answer: 80

Expert's comment: Relevance can be defined in different ways, but I do think relevance differs between domains. Selection of variables should be based on potential relevance in the context of the study (e.g., modifiable vs. non-modifiable).

Expert's answer: 60

Expert's comment: I agree with the sentiment but it doesn't really say anything useful does it?

Expert's answer: 70

Expert's comment: I think you are referring to phenotypes defined within different domains and I agree that they may be informative for different purposes. It's very unclear if they would be more or less relevant though and we might need to explain further what we mean by "different".

Expert's answer: 75

Expert's comment: I am interpreting this to mean that one domain is being used to define a phenotype. This statement differs from #8 which essentially states that different parameters from different domains should be used to define phenotypes. So while I agree with this statement, its relevance is questionable if we are to agree that using multiple parameters across domains is preferable to define a phenotype.

Expert's answer: 0

Expert's comment: Relevance is a supercharged word suggesting that one is more important than another; I do not agree that pain is more important than joint space narrowing; the escalation of pain as the “fifth vital sign” is believed to have exacerbated the opioid epidemic; I believe attention to each facet of disease is important and the elevation of subjective over objective indicators is incorrect, counterproductive and in part, has contributed to the slow pace of therapeutic advances for OA. I take issue with the term relevance.

Expert's answer: 100

Expert's comment: Absolutely

Expert's answer: 70

Expert's comment: Related to question 9, I think that the relevance should also be different. However, I expect that every separate phenotype has a different clinical relevance (in general).

Expert's answer: 80

Expert's comment: Yes, see answer 9

Expert's answer: 70

Expert's comment: Yes, but again it depends on your intention

| **Question 11** | OA in *different joints* represents different phenotypes. |
| --- | --- |
| **Mean** | 70 |
| **SD** | 17 |
| **Median** | 70 |

Expert's answer: 90

Expert's comment: Again the answer is dependent on IF the disease in different joints has different trajectory/pathobiology and therefore likely different treatment.

Expert's answer: 90

Expert's comment: IF the trajectory/pathobiology and therefore treatment are different

Expert's answer: 40

Expert's comment: This depends. For example, across joints there may be greater similarities in pathology than in functional consequences.

Expert's answer: 90

Expert's comment: Yes - phentoyping should be done separately for each of the main joints affected by OA. Some phenotypes are probably overarching and apply at different anatomical sites (e.g. a phenotype driven by central sensitisation to pain), but key phenotypes will be joint specific (e.g. varus malaligned medial tibiofemoral OA)

Expert's answer: 50

Expert's comment: Not necessarily, but maybe in some cases. Nodal hand OA may be its own phenotype, but hand OA along with knee OA in a person with metabolic syndrome is probably due to the same process as the knee OA.

Expert's answer: 80

Expert's comment: Phenotypes can be defined locally or systemically. Yet, I think that the concept of phenotypes differs between joints, in terms of predominating underlying processes and relevance of phenotypes between joints.

Expert's answer: 70

Expert's comment: Hip vs medial TFJ for example I agree. But DIPJ vs PIPJ? But if systemic treatments are to have any place in management of OA, this statement can only be true up to a point?

Expert's answer: 90

Expert's comment: And patterns of joint involvement might be informative for phenotyping.

Expert's answer: 90

Expert's comment: yes, physiopathology and risk factors are different

Expert's answer: 60

Expert's comment: This is partially true. E.g. morphometric abnormalities leading to unilateral hip OA for instance is quite distinct from nodal hand OA. However, post-traumatic OA in any joint might represent a similar pathological process and therefore phenotype.

Expert's answer: 70

Expert's comment: Controversial, needs further discussion. This will be true for some e.g. etiogenic, but maybe not for [prognostic or predictive

Expert's answer: 80

Expert's comment: I’m pretty sure that this is true. Even between the hip and knee joints I’m expected difference in phenotypes. Which is even stronger between knee and hand joints.

Expert's answer: 60

Expert's comment: This depends on the factors used for phenotype classification.

| **Question 12** | *Structural OA stages* (e.g., Kellgren and Lawrence grade 1 vs. 3) represent different phenotypes. |
| --- | --- |
| **Mean** | 35 |
| **SD** | 25 |
| **Median** | 30 |

Expert's answer: 60

Expert's comment: Structural OA stages represent a continuum of severity (stages on a single dimension) but I think of phenotypes as being multi-dimensional.

Expert's answer: 20

Expert's comment: K&L should not be used to define phenotypes. Furthermore, progression of sturcutral damage will be inherent to many different aetiological / mechanistic phenotypes. For me, phenotype thinking is fundamentally different from thinking in disease stages.

Expert's answer: 50

Expert's comment: JSN or OST might but perhaps not global grades alone, might be part of a phenotype that includes other features.

Expert's answer: 30

Expert's comment: KL grade has very little impact on clinical treatments other than arthroplasty.

Expert's answer: 50

Expert's comment: I think the underlying, driving processes vary in dominance during the OA course, but think phenotypes had best be defined for the OA course in individual patients or joints as a whole, rather then per disease stage.

Expert's answer: 20

Expert's comment: This is disease status/stage.

Expert's answer: 30

Expert's comment: a stage is not really a phenotype, patients in the same stage can be very different, the evolution from a stage to the "next" one is highly variable

Expert's answer: 60

Expert's comment: Due to its tenuous link with pain in particular and hence clinical presentation, KL grade in and of itself as a phenotype is questionable

Expert's answer: 0

Expert's comment: No, different stages of severity. It is clear that we need agreement about what constitutes a phenotype vs a stage or grade of disease. Review of grading, staging, phenotypes in other fields might help to provide clarity.

Expert's answer: 50

Expert's comment: I’m absolutely not sure whether this is true. Since K&L scores have not much impact on other variables (weak relationships) I don’t expect that the different stages also represent different phenotypes.

Expert's answer: 30

Expert's comment: I quess KL grade it not a good phenotype factor, as it it not well related to prognosis, treatment response or tailored treatment

Expert's answer: 20

Expert's comment: No, stage is not a phenotype. However, a phenotype can become more clear when you are a bit further in the disease.

| **Question 13** | *Clinical OA stages* (e.g., recent onset vs. long-standing pain) represent different phenotypes. |
| --- | --- |
| **Mean** | 50 |
| **SD** | 30 |
| **Median** | 50 |

Expert's answer: 90

Expert's comment: Persistent pain is associated with changes across a range of dimensions (pain processing, psychological, functional adaptations, life-style, sleep etc).

Expert's answer: 20

Expert's comment: Same answer as to previous question - phenotypes are not disease stages.

Expert's answer: 90

Expert's comment: probably

Expert's answer: 60

Expert's comment: This may be relevant for treatment e.g. chronic pain state versus acute pain flair.

Expert's answer: 50

Expert's comment: Similar to question 12

Expert's answer: 30

Expert's comment: Not necessarily as they may also be very heterogeneous groups

Expert's answer: 70

Expert's comment: yes because they can share similar characteristics, e.g behavior face to face with pain is different in recent-onset vs long-standing pain (this is less variable than kellgren stage)

Expert's answer: 0

Expert's comment: Since you don’t know how the “recent onset’ will evolve, I don’t see how you can say this. In fact, if by recent onset, you mean individuals with new onset radiographic OA, then I would say they have already suffered from OA for many years and did not know it or it was silent (serological).

Expert's answer: 70

Expert's comment: Phenotyping based on clinical stages has a strong interest, because the impact of the different types on intervention is to be expected. I expect that this is possible.

Expert's answer: 70

Expert's comment: This is a better factor than KL grade, as the treatment would be different in different stages

Expert's answer: 20

Expert's comment: No, stage is a phenotype.

| **Question 14** | OA populations for investigating phenotypes should consist of people in a *similar OA stage* (either clinical or structural) or analyses should at least be adjusted for that. |
| --- | --- |
| **Mean** | 60 |
| **SD** | 23 |
| **Median** | 65 |

Expert's answer: 95

Expert's comment: A I think stage is a phenotype then logically YES this needs to be accounted for when studying phenotypes

Expert's answer: 55

Expert's comment: Analyses should be controlled for disease duration but for me phenotypes are largely independent from disease stages.

Expert's answer: 30

Expert's comment: Not sure that is necessary and might limit ability to characterize phenotypes if the population is too homogeneous.

Expert's answer: 50

Expert's comment: I would like to say "no" here, but having had experience publishing in this field, several reviewer have stated this point so I think there is a general consensus that it should.

Expert's answer: 80

Expert's comment: The influence/relevance of disease stage and how this may affect results should at least be discussed. Reasons for adjusting or not adjusting for disease stage should be discussed.

Expert's answer: 80

Expert's comment: I believe this is important in order to separate phenotypes from markers of disease severity.

Expert's answer: 70

Expert's comment: In principle, I agree with this however, it gets a bit tricky when we consider studies of trajectories of subroups (of which several have been published) and it is therefore possible that during these trajectories some people change stage of disease.

Expert's answer: 60

Expert's comment: Probably

Expert's answer: 80

Expert's comment: Statistically, adjustification needs to be performed. To find differences in a homogeneous group (similar OA stage) makes it more interesting when subgroups can be found.

Expert's answer: 50

Expert's comment: Not necessarily, this depends on the phenotype factors that are used.

Expert's answer: 70

Expert's comment: Otherwise you will find clusters of stage

| **Question 15** | A presumed phenotype comprising just *a very limited number of subjects* is less relevant. |
| --- | --- |
| **Mean** | 47 |
| **SD** | 31 |
| **Median** | 40 |

Expert's answer: 30

Expert's comment: As long as its a valid phenotype with different trajectory/pathobiology and therefore treatment and outcome the number affected should not be an issue (as with any "rare disease")

Expert's answer: 80

Expert's comment: It depends. A scientifically-validated phenotype with a low prevalence might still be clinically important. However, a phenotype that is not scientifically validated and presumed to exist by observing a very limited number of subjects is likely to be less relevant.

Expert's answer: 60

Expert's comment: Partially agree, in that it would be best if we could identify the 'big hitters', but distinct phenotypes with comparatively low prevalence (e.g., inflammatory OA) can be crucial to our understanding and may provide an immediate improvement in treatment allocation and success.

Expert's answer: 15

Expert's comment: If a phenotype was strongly predictive of a poor outcome or specific treatment response, or particularly if it was associated with poor response to therapy, would be important to characterize and specifically include or exclude for example from a clinical trial.

Expert's answer: 75

Expert's comment: This depends on the subgroup. A fast progressing, severe pain group would represent an important group clinically, but a small group that is only marginally different from larger groups would not be very relevant.

Expert's answer: 80

Expert's comment: I think findings in a limited number of subjects might be of relevance to other phenotypes as well. Yet, I think that the final impact (and, with that, relevance) for clinics, trials etc. generally is less, unless they can be very well recognized and distinguished.

Expert's answer: 0

Expert's comment: Depends how serious and how amenable to effective prevention/management it is. A research strategy of breaking off small well-defined phenotypes from the heterogeneous OA population could be productive.

Expert's answer: 30

Expert's comment: A very well characterised minority could be important for targeted treatment.

Expert's answer: 30

Expert's comment: studying a rare disease is important for the patients concerned..and also to test some hypothesis

Expert's answer: 30

Expert's comment: Have to disagree here…we could find something on the rarer side but having an important effect on those who it impacts

Expert's answer: 0

Expert's comment: Strongly disagree, deep phenotyping of a small sample is more valuable than superficial phenotyping of a large sample

Expert's answer: 0

Expert's comment: Should be a combination of the 2

Expert's answer: 80

Expert's comment: Yes, for phenotyping a large number of participants is necessary to make the subgroups more stable. On a limited number of participants makes it less relevant because of the instability of results.

Expert's answer: 50

Expert's comment: In one way it is, but on the other hand, if this small subgroup can only be treated effectively with a subgroup-specific intervention that is available than this phenotype classification is highly relevant.

Expert's answer: 40

Expert's comment: On population level it might be less relevant, but for the individual it might be highly relevant

| **Question 16** | OA phenotypes derived from *data-driven* approaches should be valued more highly than those based on *expert opinion*. |
| --- | --- |
| **Mean** | 75 |
| **SD** | 19 |
| **Median** | 80 |

Expert's answer: 85

Expert's comment: I am interpreting data-driven here to mean 'based on the analysis of data and interpreted clinically', rather than to mean 'based on data and analyses that are not informed by clinical interpretability'.

Expert's answer: 60

Expert's comment: IF (and it's a big if) the data quality is excellent, then yes. As in any other field of medical science.

Expert's answer: 60

Expert's comment: There is certainly value in expert opinion and clinical experience, but it is also important to consider novel phenotypes that might not be obvious in clinical practice, and to be able to characterize phenotypes based on data which might not always be available in every clinical situation (but could be in research or a trial).

Expert's answer: 80

Expert's comment: Expert opinion could be biased. A data-driven approach interpreted by experts would be an optimal process.

Expert's answer: 80

Expert's comment: When performed adequately, I think data-driven approaches are important for making progress in the field.

Expert's answer: 30

Expert's comment: These things need to work together but I find it possible that genetic insights may alter our thinking about OA phenotypes in ways not anticipated by expert clinical opinion

Expert's answer: 80

Expert's comment: Data-driven approaches reduce subjectivity.

Expert's answer: 100

Expert's comment: I agree but we lack sufficient evidence to rely upon it independent of expert opinion

Expert's answer: 50

Expert's comment: Should be a combination of the 2

Expert's answer: 80

Expert's comment: Expert opinions are important in the design of studies, but in the end phenotyping is determined by the large study population (and by data driven approaches).

Expert's answer: 50

Expert's comment: Not really. I think that both data-driven and expert opinion based phenotypes should be first tested in the real world (clinical practice), to test whether these phenotypes do exist.

Expert's answer: 40

Expert's comment: Phenotypes defined based on both approaches should be examined on whether there are prognostic or therapeutic implications

**Transition round 2 to 3**

**Statements round 1:**

- Patient subgroups with different longitudinal trajectories of an OA-related variable (e.g., pain, joint space width) represent OA phenotypes (*score:* mean 59, SD 27, median 60).

- Patient subgroups with different predominating OA-related variables (e.g., synovitis vs. osteophytes) represent OA phenotypes (*score:* mean 72, SD 17, median 70).

- Patient subgroups differing with regard to response to a particular treatment represent different OA phenotypes (*score*: mean 69, SD 23, median 60).

- Patient subgroups differing with regard to prognostic factors represent OA phenotypes (*score:* mean 62, SD 26, median 60).

- Patient subgroups differing with regard to etiological backgrounds represent OA phenotypes (*score:* mean 66, SD 25, median 70).

- OA subtype/subgroup is synonymous with OA phenotype (*score:* mean 63, SD 30, median 60).

- *Many experts think that trajectories, predominating OA features, treatment responses, and prognostic risk factors each capture different aspects of the concept of phenotypes, but that they are not synonymous.*
- *Multiple experts think subgroups are not similar to subtypes. Most think subtyping comes closer to phenotyping, because it implies shared underlying disease mechanisms within a subtype, as opposed to subgroups that can be defined by any criterion or even by chance.*

**Statements round 2:**

1. OA phenotypes are subtypes of OA that share, or can reasonably be assumed to share, distinct underlying functional or pathobiological mechanisms (*score:* mean 81, SD 17, median 80).

2. OA phenotypes can become apparent in differences in risk factors, nature and extent of symptoms and signs, disease trajectory, and/or responsiveness to particular treatments or treatment in general (*score:* mean 82, SD 15, median 75).

- *One expert proposes to use “endotypes” rather than “phenotypes”.*
- *Some experts question the use of “functional … mechanisms”. Another emphasizes that biopsychosocial mechanisms should be included in the definition, in addition to the biomedical approach.*
- *One expert advises to add “prognostic factors” to the second statement.*
- *One expert argues that external factors also affect responsiveness to treatment, in addition to the internal factors that together make up phenotypes.*
- *One expert thinks the current definition allows too much room for different classifications of phenotypes (e.g. treatment response phenotypes, prognostic phenotypes, etc.)*

**Responses:**

- Using the term “endotypes” may indeed be more appropriate than “phenotypes”. However, the term “phenotypes” is used so much in current literature already that it may be confusing to replace this term.

- The term “functional” was meant to indicate other factors affecting the function rather than the structure of tissues.

- We do acknowledge that other (external) factors than the phenotype may determine clinical outcome, but don’t see how to incorporate this in the definition of OA phenotypes.

**New statements:**

- OA phenotypes are subtypes of OA that share, or can reasonably be assumed to share, distinct underlying pathobiological mechanisms and their structural and functional consequences.

- OA phenotypes can become apparent in differences in risk factors, prognostic factors, nature and extent of symptoms and signs, disease trajectory, and/or responsiveness to particular treatments or treatment in general.

**Statements round 1:**

- Overlap between phenotypes is to be expected (*score:* mean 78, SD 19, median 80).
- Patient subgroups should preferably be defined using parameters from different OA domains (e.g., structural, functional, psychological) (*score:* mean 70, SD 22, median 70).
- Not all domains are equally efficient in separating OA phenotypes (e.g., pain vs. radiographic joint space narrowing) (*score:* mean 80, SD 17, median 80).
- The relevance of OA phenotypes defined from different domains is different (e.g., pain vs. radiographic joint space narrowing) (*score:* mean 67, SD 23, median 70).
- *Multiple experts express a preference of clinically relevant and/or modifiable OA-related variables to be used in phenotype classification (e.g. synovitis and pain over osteophytes and K&L grade).*
- *Multiple experts do not fully understand or do object to the terms “relevance” and “efficiency” of OA-related variables and domains. These terms, after all, depend on the context of the proposed phenotype classification.*
- *Experts do acknowledge the complexity of OA, but not all agree that phenotypes should be classified from OA-related variables from different domains. Multiple experts also emphasize that such an approach would have to be very comprehensive and be very costly. They also doubt whether it would be feasible at the moment, considering limitations in our ability to fully capture every domain appropriately.*

**Statements round 2:**

3. A system to classify OA patients for one or more phenotypes should consist of input variables that together reflect (the likelihood of) the presence of one or more functional or pathobiological mechanisms in these patients (*score:* mean 81, SD 12, median 75).

4. To ascertain that OA phenotype classification systems could eventually influence decision-making in clinical trials and practice, the potentially identified phenotype(s) should differ from others in terms of disease-driving factors and/or outcomes, e.g. disease trajectory and/or responsiveness to particular treatments or treatment in general, etc. (*score:* mean 81, SD 15, median 80)

5. As OA is a complex, multidimensional disease, it is likely that for classification systems to be effective, input variables should together assess OA from different perspectives (e.g. physical examination, imaging, biochemical markers, etc.) (*score:* mean 76, SD 19, median 80).

6. Phenotype classification systems can be developed in different contexts and with different goals. Therefore, there could be multiple OA phenotype classification systems that would be used either separately or in combination, e.g., systems that relate to prognosis and systems that relate to treatment response (*score:* mean 75, SD 19, median 70).

- *One expert thinks the fourth statement looks too similar to the second statement.*
- *Some experts come back to the point that assessing OA from different perspectives may be too costly or infeasible. Some also question the need.*
- *Some experts think combining structural and clinical parameters in classification systems may be difficult as they usually are not highly correlated.*
- *One expert opposes to the use of multiple classification systems, scoring 10.*

**Response:**

The difference between the fourth and second statement lies in the fact that not all differences between the consequences of phenotypes might be clinically relevant in extent and/or nature.

**New statements:**

- A system to classify OA patients for one or more phenotypes should consist of input variables that together reflect (the likelihood of) the presence of one or more pathobiological and biopsychosocial mechanisms and their structural and/or functional consequences in these patients.

- To ascertain that OA phenotype classification systems could eventually influence decision-making in clinical trials and practice, the potentially identified phenotype(s) should differ from others in terms of clinically relevant disease-driving factors and/or outcomes, e.g. disease trajectory and/or responsiveness to particular treatments or treatment in general, etc.

- Classification systems could use one or more input variables from either one or more domains (e.g., imaging, biochemical, pain, etc.) to identify a clinically relevant OA phenotype or phenotypes.

- Phenotype classification systems can be developed in different contexts (e.g., research vs. daily practice) and with different goals (e.g., supporting treatment decisions vs. selection of patients for studies). Therefore, there could be multiple OA phenotype classification systems that would be used either separately or in combination, e.g., systems that relate to prognosis and systems that relate to treatment response.

**Statements round 1:**

- Structural OA stages (e.g., Kellgren and Lawrence grade 1 vs. 3) represent different phenotypes (*score:* mean 35, SD 25, median 30)
- Clinical OA stages (e.g., recent onset vs. long-standing pain) represent different phenotypes (*score:* mean 50, SD 30, median 50).
- OA populations for investigating phenotypes should consist of people in a similar OA stage (either clinical or structural) or analyses should at least be adjusted for that (*score:* mean 60, SD 23, median 65).
- *Most experts agree that disease stages are not similar to phenotypes.*
- *Many experts think disease stage is relevant for phenotype classification, but they differ in their argumentation.*

**Statements round 2:**

7. Disease stage is likely to be relevant for OA phenotyping, as disease stages in themselves might already differ in the predominance of etiological pathways and our ability to capture their typical characteristics (e.g. minimal vs. extensive cartilage changes, preclinical metabolic derangements vs. overt structural disease, etc.) (*score:* mean 66, SD 22, median 70).

8. It should, however, also be acknowledged that the nature and course of disease stages may differ between patients and phenotypes and that our methods for disease staging have their limitations. Reasons to adjust or not adjust analyses for disease stage should be weighed for every study (*score:* mean 80, SD 16, median 80).

- *Some authors seem to understand that the statements say that disease stages are phenotypes, like one did in the first round.*
- *One expert opposes to the word “relevant”.*
- *One expert adds that one could also look for interaction with rather than adjusting for disease stage.*
- *Some experts think these statements had better be combined or that one may replace the other.*
- *Some experts say it’s hard to define disease stages.*

**Responses:**

- We did not mean to say disease stages are similar to phenotypes. We aimed to say that disease stage might affect the results from phenotype research, as they themselves probably differ in nature and extent of pathobiological and biopsychosocial mechanisms.
- The eighth statement confirms that defining disease stage is not straightforward.

**New statements:**

- Differences in disease stage may affect the results from OA phenotyping studies, as disease stages in themselves might already differ in the predominance of etiological pathways and our ability to capture their typical characteristics (e.g. minimal vs. extensive cartilage changes, preclinical metabolic derangements vs. overt structural disease, etc.).

- It should, however, also be acknowledged that the nature and course of disease stages may differ between patients and phenotypes and that our methods for disease staging have their limitations. Reasons to take or not take disease stage into account in the analyses (e.g., to adjust for confounding or look for interaction) should be weighed for every study.

**Statement round 1:**

A presumed phenotype comprising just a very limited number of subjects is less relevant (*score:* mean 47, SD 31, median 40).

- *Most experts, some conditionally, acknowledge that a phenotype comprising just a limited number of subjects can be important.*

**Statement round 2:**

9. A phenotype classification category comprising just a very limited part of the population as a whole can well be informative, particularly when it is consistently and clearly distinguishable from the others and when it impacts decision making in clinical trials or practice (*score:* mean 88, SD 10, median 85).

- *All comments are in support of the statement.*

**This statement is closed.**

**Statement round 1:**

OA in different joints represents different phenotypes (*score:* mean 70, SD 17, median 70).

- *Most experts agree that functional or pathobiological mechanisms may to a certain extent be similar between joints, but that joint-specific mechanisms will play an important role too, e.g. menisci in the knee and femoral head shape in the hip. Moreover, some mechanisms may affect single joints, e.g., trauma, while others will affect multiple joints, e.g. metabolic factors.*
- *Multiple experts mention that phenotype classification systems can be set up either per joint type or systemically, for multiple joints in one patient, depending on the context.*

**Statements round 2**

10. Phenotype classification systems for individual joints can probably not be translated directly to other joints. Although different joints may share common functional or pathobiological OA mechanisms, their consequences probably differ between joints and joint-specific factors (e.g. menisci, femoral head shape) also play a role (*score:* mean 72, SD 19, median 75).

11. Phenotype classification systems can be set up either per joint type or systemically, for multiple joints in one patient, depending on their intended context and goal (*score:* mean 78, SD 14, median 80).

- *Some experts say that phenotypes may or may not differ between joints, depending on the phenotype.*
- *One expert thinks phenotypes on a systemic level are of no use.*
- *One expert doesn’t understand the use of “context and goal”.*
- *These two statements had better be combined.*

**New statement**

- Some components of pathobiological and biopsychosocial OA mechanisms may be similar between different joints such as knee and hip (e.g., synovitis, central pain perception), while others may differ (e.g., menisci, femoral head shape). When extrapolating findings in one joint to another joint, it should be explained why this would be appropriate.

- In line with this, phenotype classification systems can be designed for individual joints or systemically, for multiple joints in one patient, depending on the pathobiological and biopsychosocial mechanism that is under study and the goal of the study (e.g., study OA impact in an individual versus predict cartilage loss in a knee).

**Statement round 1:**

OA phenotypes derived from data-driven approaches should be valued more highly than those based on expert opinion (*score:* mean 75, SD 19, median 80).

- *Most experts tend to agree with the statement, but multiple experts emphasize the need for high-quality data and statistics for such approaches and the importance for clinical validity, relevance and applicability.*

**Statement round 2:**

12. Data-driven approaches for constructing phenotype classification systems are generally preferable over expert opinion-based approaches, as long as they are performed using high-quality data and statistics and with attention to clinical validity, relevance and applicability (*score:* mean 84, SD 15, median 80).

- *One expert suggests adding “reproducibility”.*
- *One expert suggests adding “expert opinion”.*

**New statement:**

Data-driven approaches for constructing phenotype classification systems are generally preferable over expert opinion-based approaches, as long as they are performed using high-quality data and appropriate statistics, are reproducible, and have clinical validity, relevance and applicability as judged by experts in the field.

**Results Delphi round 2**

| **Question 1** | OA phenotypes are subtypes of OA that share, or can reasonably be assumed to share, distinct underlying functional or pathobiological mechanisms. |
| --- | --- |
| **Mean** | 81 |
| **SD** | 17 |
| **Median** | 80 |

Expert's answer: 50

Expert's comment: The term endotype is more specific to shared distinct pathobiological mechanisms than phenotype.

Expert's answer: 50

Expert's comment: i don't think "functional mechanisms" is a clear concept and may be not the right words. "...distinct physiopathological mechanisms or characteristics". the difference between groups and types is interesting to keep but what is interesting to study is not only disease mecanisms but also the evolution: i guess this is what you mean with "functional mechanisms"

Expert's answer: 65

Expert's comment: Phenotypes (simple identifiable characteristic) do not necessarily share pathobiological mechanisms - BUT it is probably more likely than is different phenotypes. I think the only "phenotypes" we should recognize ARE those that cluster similar pathobiology.

Expert's answer: 100

Expert's comment: I agree!

Expert's answer: 50

Expert's comment: This definition is quite biomedical, whereas the clinical presentation is a biopsychosocial phenomenon. By functional, I assume you are referring to the activity limitations (functional consequences) of the condition. The evidence indicates that the activity limitations of OA are to some extent influenced by the cognitions and emotions of the person.

Expert's answer: 95

Expert's comment: I think this is a far closer representation that the previous round.

Expert's answer: 60

Expert's comment: Can we clarify what is the meaning intended by the word functional (mechanism)?

Expert's answer: 80

Expert's comment: Distinction between functional and pathobiological mechanisms is necessary but can be part of analysis process.

| **Question 2** | OA phenotypes can become apparent in differences in risk factors, nature and extent of symptoms and signs, disease trajectory, and/or responsiveness to particular treatments or treatment in general. |
| --- | --- |
| **Mean** | 82 |
| **SD** | 15 |
| **Median** | 75 |

Expert's answer: 90

Expert's comment: this is what we want to define and study: characterizing groups even if the word phenotype is often used to classify patients with common disease mechanisms, i think this is also important to include trajectories and responsiveness to treatment...

Expert's answer: 70

Expert's comment: Largely agree, but it leaves some room for confusion and further subdivision into 'treatment phenotypes', 'aetiological phenotypes' etc. Maybe a more strict definition would be more beneficial?

Expert's answer: 75

Expert's comment: I think it could be argued that phenotypes are more baseline/inception characteristics. Responsiveness to particular treatments or treatment in general may be influenced by extrinsic, not just intrinsic factors. Also, state characteristics e.g., adherence or self-efficacy may influence responsiveness and be indicative that different treatments are required as opposed to a phenotype.

Expert's answer: 40

Expert's comment: In amalgamating the previous statements into this new one it seems that the idea of prognostic factors has been lost/replaced by risk factors. Given that the two have different meanings and applications depending on the population being studied, I think it important to keep them both.

Expert's answer: 60

Expert's comment: This is too vague. It’s not clear to me what the rationale is behind this question.

| **Question 3** | A system to classify OA patients for one or more phenotypes should consist of input variables that together reflect (the likelihood of) the presence of one or more functional or pathobiological mechanisms in these patients. |
| --- | --- |
| **Mean** | 81 |
| **SD** | 12 |
| **Median** | 75 |

Expert's answer: 90

Expert's comment: i agree (except for the term functional mechanisms)

Expert's answer: 100

Expert's comment: Completely agree

Expert's answer: 80

Expert's comment: I agree, but just remembering that phenotypes may be also identified retrospectively, i.e. starting from the outcome and then looking back to look for risk factors or other factors that may explain the differences in, for example, disease progression or treatment response

Expert's answer: 60

Expert's comment: Only if functional or pathobiological mechanisms are the things most valued. For example, pathobiological mechanisms in OA may only partially explain clinical presentation and treatment response.

Expert's answer: 95

Expert's comment: I agree that the confluence of variables is likely to be more important than a single trait characteristic.

Expert's answer: 60

Expert's comment: Not convinced that this will always be a clinically relevant or feasible approach. I also agree as pointed out by someone that this could vary depending on the objective of how the phenotypes are being used e.g. analyze health state and/or its evolution, vs. personalize a treatment

Expert's answer: 70

Expert's comment: I am not sure what this is actually saying.

Expert's answer: 80

Expert's comment: Yes, this reflects exactly what I mean.

| **Question 4** | To ascertain that OA phenotype classification systems could eventually influence decision-making in clinical trials and practice, the potentially identified phenotype(s) should differ from others regarding disease-driving factors and/or outcomes, e.g. disease trajectory and/or responsiveness to particular treatments or treatment in general, etc. |
| --- | --- |
| **Mean** | 81 |
| **SD** | 15 |
| **Median** | 80 |

Expert's answer: 90

Expert's comment: Yes, a phenotype classification that is not clinically useful is pointless - and the classification can only be useful if it meaningfully distinguishes between patients in which treatment option has the highest probability of working for them.

Expert's answer: 40

Expert's comment: Wordy repeat of clause already captured in Q2?

Expert's answer: 100

Expert's comment: Absolutely

Expert's answer: 70

Expert's comment: See my previous comment regarding responsiveness to treatment. Provided that the treatment was implemented as designed, then I think this statement holds merit, but given the variance in implementation I think it may be reasonable to discuss latent characteristics here or more general patterns of trajectories.

Expert's answer: 60

Expert's comment: I don’t know that we know enough at this point to be able to confidently say one way or another. It may be that certain phenotypes e.g. defined by pain are useful for determining both trajectories, prognosis or treatment

Expert's answer: 80

Expert's comment: Yes, particularly in decision-making processes.

| **Question 5** | As OA is a complex, multidimensional disease, it is likely that for classification systems to be effective, input variables should together assess OA from different perspectives (e.g. physical examination, imaging, biochemical markers, etc.). |
| --- | --- |
| **Mean** | 76 |
| **SD** | 19 |
| **Median** | 80 |

Expert's answer: 75

Expert's comment: This makes sense, but as noted in the first round has implications for cost and complexity of data collection. If a phenotype was based only on one or two of these domains but was associated with outcomes, that could still be very useful.

Expert's answer: 80

Expert's comment: One of the major challenges overcome the factor that we only treat symptoms. Phenotypes should most like describe the underlining mechanism that leads to the symptoms and to provide better tool for treatment to target/cause

Expert's answer: 30

Expert's comment: i think this is difficult to combine clinical and Imaging data as they are not highly correlated and do not respond alongside the answer might depend on the phenotype : with "outcome phénotypes" (trajectories or response to treatment), i would say no, if the phenotype is used to predict an outcome, i would say yes. different perspectives should be studied but not together effective: usefull ?

Expert's answer: 80

Expert's comment: Yes - some phenotypes can only be defined with input from multiple perspectives.

Expert's answer: 80

Expert's comment: I agree although this may be challenging from the analytical point of view. I think we should consider all dimensions but differentiating what are the phenotypes of pain/function vs. structural phenotypes

Expert's answer: 60

Expert's comment: But classification systems are broader than phenotyping. It is unrealistic to require phenotyping to fully embrace the complex multidimensional nature of OA.

Expert's answer: 100

Expert's comment: Absolutely

Expert's answer: 30

Expert's comment: A “magic bullet” test could be sufficient perhaps to identify some forms, eg based on a genetic polymorphism like cancer or low selenium for instance so I disagree that it necessarily will require different domains of testing to classify specific types of OA; perhaps one could say ability to classify ALL OA would require lots of different domains of testing/biomarkers.

Expert's answer: 50

Expert's comment: It isn’t clear to me how this statement differs from #3. Do these different perspectives represent the evaluation of different mechanisms?

Expert's answer: 90

Expert's comment: Need to be a little clearer that these input variables , or at least most, will need to be in each phenotype, rather than individual phenotypes for each input variable

Expert's answer: 80

Expert's comment: Yes, totally in agreement with this.

| **Question 6** | Phenotype classification systems can be developed in different contexts and with different goals. Therefore, there could be multiple OA phenotype classification systems that would be used either separately or in combination, e.g., systems that relate to prognosis and systems that relate to treatment response. |
| --- | --- |
| **Mean** | 75 |
| **SD** | 19 |
| **Median** | 70 |

Expert's answer: 90

Expert's comment: with different goals: yes in different contexts: this is may be not enough specific (the term)

Expert's answer: 10

Expert's comment: Please no - this would get messy really quickly and defeat its purpose.

Expert's answer: 60

Expert's comment: That relates to my previous answer but in the context of symptoms vs structure. I think it is important to avoid confusion in the sense that prognostic factors and predictors of treatment response may help to identify phenotypes but are not necessarily phenotypes

Expert's answer: 60

Expert's comment: Feels inclusive but perhaps we need some examples of what might be 'successful' different phenotype classification systems to illustrate this

Expert's answer: 100

Expert's comment: Prognostic phenotypes may describe combinations of factors that affect outcome regardless of treatment. Treatment effect modifier phenotypes are specific to treatments. They are different and co-exist.

Expert's answer: 60

Expert's comment: I agree that different classification systems make sense, but I question the utility of such a system. The optimal situation would be to have single/few systems. The likelihood of that happening is small, given the complexity of the disease.

Expert's answer: 75

Expert's comment: Yes, and I think important to note the ‘separately or in combination’ aspect.

Expert's answer: 70

Expert's comment: I’m not fully convinced that phenotype classifications should be used I different ways.

| **Question 7** | Disease stage is likely to be relevant for OA phenotyping, as disease stages in themselves might already differ in the predominance of etiological pathways and our ability to capture their typical characteristics (e.g. minimal vs. extensive cartilage changes, preclinical metabolic derangements vs. overt structural disease, etc.). |
| --- | --- |
| **Mean** | 66 |
| **SD** | 22 |
| **Median** | 70 |

Expert's answer: 60

Expert's comment: Not sure on this one. Might disease stage be a proxy (and potentially a poor one) for some other risk factor that could better define a phenotype?

Expert's answer: 50

Expert's comment: Stage could be an important variable. The downfall with stage is that it is a snap shot of the "moment". It does not provide info on how it got or what got it to that stage or what comes next. However it may be important for understanding how to treat.

Expert's answer: 40

Expert's comment: Stages can have an impact on response to treatment but they are not phenotypes. some phenotypes can be associated with some stages they are relevant to analyze and interpret results when we will use phenotypes in studies but "be relevant for OA phenotyping" : i am not sure this is the good wording

Expert's answer: 30

Expert's comment: I think this needlessly complicates the issue - each underlying mechanism may result in different progression to the disease with different stages, but the bottom line is still the underlying mechanism which we aim to describe through a phenotype classification.

Expert's answer: 85

Expert's comment: It might be a "sub-phenotype" rather than a phenotype on its own - e.g. might be "early" "late" inflammatory OA or metabolic OA. So it sub-divides a phenotype BUT the 2 late stage phenotypes maybe distinct from each other (i.e. it matters how you got to late stage disease"

Expert's answer: 60

Expert's comment: I agree that different disease stages is important for several reasons. However, I don't think they are different phenotypes but rather subgroups that may exist even within the same phenotype

Expert's answer: 70

Expert's comment: As per round 1 feedback - may need to be clear what is meant by 'relevant'

Expert's answer: 85

Expert's comment: Yes, I think staging is a potentially useful factor to input to phenotypes for the reasons you state.

Expert's answer: 60

Expert's comment: Given the dissociation between structural damage and clinical features, I'm skeptical that phenotyping would gain from including disease stages.

Expert's answer: 0

Expert's comment: This still makes little sense to me. To make sense it would be necessary to qualify this, for instance, “differences of disease stage severity after accounting for other risk factors (age, gender, BMI, etc) may be relevant for OA phenotyping.”

Expert's answer: 85

Expert's comment: Need to combine with 8, see below

Expert's answer: 70

Expert's comment: Pretty vague to me. What is exactly meant with “disease stage”?

Expert's answer: 60

Expert's comment: This simply is staging; I am not sure I would call this phenotyping in the real meaning of the word. On the other hand this is a very useful subtyping in cancer treatment and prognosis.

| **Question 8** | It should, however, also be acknowledged that the nature and course of disease stages may differ between patients and phenotypes and that our methods for disease staging have their limitations. Reasons to adjust, or not adjust analyses for disease stage should be considered for every study. |
| --- | --- |
| **Mean** | 80 |
| **SD** | 16 |
| **Median** | 80 |

Expert's answer: 90

Expert's comment: interactions can also be studied.

Expert's answer: 20

Expert's comment: Similar to previous question - this is a needless complication for me.

Expert's answer: 90

Expert's comment: see comments in previous question

Expert's answer: 90

Expert's comment: Relates to my comment to the previous statement

Expert's answer: 85

Expert's comment: Agree

Expert's answer: 100

Expert's comment: Yes. I think this is a reasonable alternative.

Expert's answer: 80

Expert's comment: This relates to comments in Q7.

Expert's answer: 80

Expert's comment: This needs to be incorporated into 7, otherwise 7 can be used in isolation without these limitations.

Expert's answer: 80

Expert's comment: Yes, but first to make clear what is meant with the different disease stages. This is a topic on its self.

Expert's answer: 60

Expert's comment: A difficult matter

| **Question 9** | A phenotype classification category comprising just a very limited part of the population as a whole can well be informative, particularly when it is consistently and clearly distinguishable from the others and when it impacts decision making in clinical trials or practice. |
| --- | --- |
| **Mean** | 88 |
| **SD** | 10 |
| **Median** | 85 |

Expert's answer: 90

Expert's comment: I think it is often the case that we are looking for small effects in big groups when we might be better served to identify large effects in small identifiable populations where we might have a greater impact on disease.

Expert's answer: 80

Expert's comment: Yes - rare phenotypes should not be ignored purely based on their prevalence.

Expert's answer: 95

Expert's comment: I do not think that the size of the group should matter. Rather, the magnitude of differentiation between groups should be important.

Expert's answer: 70

Expert's comment: It will have only impact that specific part of the population. Whether it can be used for other parts of the populations is discussable.

| **Question 10** | Phenotype classification systems for individual joints can probably not be translated directly to other joints. Although different joints may share common functional or pathobiological OA mechanisms, their consequences probably differ between joints and joint-specific factors (e.g. menisci, femoral head shape) also play a role. |
| --- | --- |
| **Mean** | 72 |
| **SD** | 19 |
| **Median** | 75 |

Expert's answer: 50

Expert's comment: It depends. Some phenotypes are likely driven by systemic factors that affect all joints while others will be limited to weight bearing joints or just one biomechanically predisposed joints. Not sure about this blanket statement.

Expert's answer: 50

Expert's comment: I partly agree. For imaging and anatomic assessments this may be true, but I blive that dependent on the variables in the phenotype composite, some of the underlining mechnism will be translationable and should be used for targeted treatment. Say if X and Y is present then you treat with A independently of the diagnosis (hence diseased joint)

Expert's answer: 40

Expert's comment: in some cases different joints could be combined

Expert's answer: 60

Expert's comment: Underlying mechanisms will be part joint-specific and part systemic - so we should have both.

Expert's answer: 90

Expert's comment: Yes, and the patient's perceived threat of the condition and its functional consequences will vary across body locations.

Expert's answer: 75

Expert's comment: I think it would be reasonable to assume that there will be whole-of-person characteristics that would be shared between phenotypes of individual joints.

Expert's answer: 30

Expert's comment: Not so sure, if inflammation is a general etiologic factor for progression then treatment of inflammation could be relevant for OA at multiple joint sites.

Expert's answer: 70

Expert's comment: “can probably not be translated directly to other joints”. Thi is too strong. “Probably” should be substituted with “may”

Expert's answer: 80

Expert's comment: I totally agree that phenotyping is joint specific.

Expert's answer: 50

Expert's comment: For some dimensions like pain it can, but for others dimensions not

| **Question 11** | Phenotype classification systems can be set up either per joint type or systemically, for multiple joints in one patient, depending on their intended context and goal. |
| --- | --- |
| **Mean** | 78 |
| **SD** | 14 |
| **Median** | 80 |

Expert's answer: 90

Expert's comment: This sounds better than the last one.

Expert's answer: 90

Expert's comment: I believe we ahould see it also coming into the digital health era. We should not be too focused on a system that can be used with pen and paper but instead a system (in the future) that are part a bioinfomatic platform. Thus the system could most likely be multi dimentional. I aware that this is not tomorrow, but the day after tomorrow

Expert's answer: 75

Expert's comment: As per the previous statement, we will have phenotypes that are indicative of systemic mechanisms and phenotypes that indicate joint-specific mechanisms - we need to do both.

Expert's answer: 50

Expert's comment: I'm not sure I understand this question

Expert's answer: 70

Expert's comment: Phrasing coul be improved. 'set up', 'per'

Expert's answer: 50

Expert's comment: I am reserved about doing this for OA at a systemic level, as I can't think of a context or goal where this would be ideal.

Expert's answer: 75

Expert's comment: I think a multi-joint involvement versus a single joint involvement may be an important pathobiological feature of a phenotype. As such, it would be ideal if these could be incorporated into a single phenotyping classification system.

Expert's answer: 90

Expert's comment: This only makes sense if you change the wording of 10 as suggested above.

Expert's answer: 60

Expert's comment: Depends on the context and aim. This is not clear to me.

| **Question 12** | Data-driven approaches for constructing phenotype classification systems are generally preferable over expert opinion-based approaches, as long as they are performed using high-quality data and statistics and with attention to clinical validity, relevance and applicability. |
| --- | --- |
| **Mean** | 84 |
| **SD** | 15 |
| **Median** | 80 |

Expert's answer: 90

Expert's comment: Evidence-based medicine: high-quality scientific evidence trumps expert opinion.

Expert's answer: 90

Expert's comment: Yes and as long as they are clinically interpretable and plausible

Expert's answer: 85

Expert's comment: Minor rephrase suggested - 'and appropriate statistical analysis' and think we should incorporate also the critical importance of reproducibility of findings

Expert's answer: 80

Expert's comment: There is no reason why this approach could not consider qualitative data as well

Expert's answer: 40

Expert's comment: Not in isolation. Data driven, supported by expert opinion would be ideal

Expert's answer: 80

Expert's comment: High quality data and statistics are needed: including extensive datafiles.

**Transition round 3 to 4**

**Statements round 1:**

- Patient subgroups with different longitudinal trajectories of an OA-related variable (e.g., pain, joint space width) represent OA phenotypes (*score:* mean 59, SD 27, median 60).

- Patient subgroups with different predominating OA-related variables (e.g., synovitis vs. osteophytes) represent OA phenotypes (*score:* mean 72, SD 17, median 70).

- Patient subgroups differing with regard to response to a particular treatment represent different OA phenotypes (*score*: mean 69, SD 23, median 60).

- Patient subgroups differing with regard to prognostic factors represent OA phenotypes (*score:* mean 62, SD 26, median 60).

- Patient subgroups differing with regard to etiological backgrounds represent OA phenotypes (*score:* mean 66, SD 25, median 70).

- OA subtype/subgroup is synonymous with OA phenotype (*score:* mean 63, SD 30, median 60).

- *Many experts think that trajectories, predominating OA features, treatment responses, and prognostic risk factors each capture different aspects of the concept of phenotypes, but that they are not synonymous.*
- *Multiple experts think subgroups are not similar to subtypes. Most think subtyping comes closer to phenotyping, because it implies shared underlying disease mechanisms within a subtype, as opposed to subgroups that can be defined by any criterion or even by chance.*

**Statements round 2:**

1. OA phenotypes are subtypes of OA that share, or can reasonably be assumed to share, distinct underlying functional or pathobiological mechanisms (*score:* mean 81, SD 17, median 80).

2. OA phenotypes can become apparent in differences in risk factors, nature and extent of symptoms and signs, disease trajectory, and/or responsiveness to particular treatments or treatment in general (*score:* mean 82, SD 15, median 75).

- *One expert proposes to use “endotypes” rather than “phenotypes”.*
- *Some experts question the use of “functional … mechanisms”. Another emphasizes that biopsychosocial mechanisms should be included in the definition, in addition to the biomedical approach.*
- *One expert advises to add “prognostic factors” to the second statement.*
- *One expert argues that external factors also affect responsiveness to treatment, in addition to the internal factors that together make up phenotypes.*
- *One expert thinks the current definition allows too much room for different classifications of phenotypes (e.g. treatment response phenotypes, prognostic phenotypes, etc.)*

**Responses:**

- Using the term “endotypes” may indeed be more appropriate than “phenotypes”. However, the term “phenotypes” is used so much in current literature already that it may be confusing to replace this term.

- The term “functional” was meant to indicate other factors affecting the function rather than the structure of tissues.

- We do acknowledge that other (external) factors than the phenotype may determine clinical outcome, but don’t see how to incorporate this in the definition of OA phenotypes.

**Statements round 3:**

1. OA phenotypes are subtypes of OA that share, or can reasonably be assumed to share, distinct underlying pathobiological mechanisms and their structural and functional consequences (*score:* mean 82, SD 13, median 80).
2. OA phenotypes can become apparent in differences in risk factors, prognostic factors, nature and extent of symptoms and signs, disease trajectory, and/or responsiveness to particular treatments or treatment in general (*score:* mean 89, SD 9, median 80).

- *Some experts say that “biopsychosocial mechanisms” should have been included in statement 1, as was done for some other statements in round 3.*
- *Some experts think “biopsychosocial mechanisms” should also be incorporated in the statements, while others don’t. It would be too non-specific and not combine well with the “pathobiological mechanism” approach.*

**Responses:**

- We agree that the term “pathobiological” on its own too much assumes that consequences from OA can all be explained from biological mechanisms in and around the joint. We also agree with other authors in that “biopsychosocial” may be too wide. We, therefore, propose using “pain mechanisms” instead.

- We apologize for omitting “biopsychosocial mechanisms” from statement 1. It should have been included in this statement as well.

**Statement round 4**

1. OA phenotypes are subtypes of OA that share distinct underlying pathobiological and pain mechanisms and their structural and functional consequences. (shortened)

**The previous statement 2 is closed:** OA phenotypes can become apparent in differences in risk factors, prognostic factors, nature and extent of symptoms and signs, disease trajectory, and/or responsiveness to particular treatments or treatment in general.

**Statements round 1:**

- Overlap between phenotypes is to be expected (*score:* mean 78, SD 19, median 80).
- Patient subgroups should preferably be defined using parameters from different OA domains (e.g., structural, functional, psychological) (*score:* mean 70, SD 22, median 70).
- Not all domains are equally efficient in separating OA phenotypes (e.g., pain vs. radiographic joint space narrowing) (*score:* mean 80, SD 17, median 80).
- The relevance of OA phenotypes defined from different domains is different (e.g., pain vs. radiographic joint space narrowing) (*score:* mean 67, SD 23, median 70).
- *Multiple experts express a preference of clinically relevant and/or modifiable OA-related variables to be used in phenotype classification (e.g. synovitis and pain over osteophytes and K&L grade).*
- *Multiple experts do not fully understand or do object to the terms “relevance” and “efficiency” of OA-related variables and domains. These terms, after all, depend on the context of the proposed phenotype classification.*
- *Experts do acknowledge the complexity of OA, but not all agree that phenotypes should be classified from OA-related variables from different domains. Multiple experts also emphasize that such an approach would have to be very comprehensive and be very costly. They also doubt whether it would be feasible at the moment, considering limitations in our ability to fully capture every domain appropriately.*

**Statements round 2:**

3. A system to classify OA patients for one or more phenotypes should consist of input variables that together reflect (the likelihood of) the presence of one or more functional or pathobiological mechanisms in these patients (*score:* mean 81, SD 12, median 75).

4. To ascertain that OA phenotype classification systems could eventually influence decision-making in clinical trials and practice, the potentially identified phenotype(s) should differ from others in terms of disease-driving factors and/or outcomes, e.g. disease trajectory and/or responsiveness to particular treatments or treatment in general, etc. (*score:* mean 81, SD 15, median 80)

5. As OA is a complex, multidimensional disease, it is likely that for classification systems to be effective, input variables should together assess OA from different perspectives (e.g. physical examination, imaging, biochemical markers, etc.) (*score:* mean 76, SD 19, median 80).

6. Phenotype classification systems can be developed in different contexts and with different goals. Therefore, there could be multiple OA phenotype classification systems that would be used either separately or in combination, e.g., systems that relate to prognosis and systems that relate to treatment response (*score:* mean 75, SD 19, median 70).

- *One expert thinks the fourth statement looks too similar to the second statement.*
- *Some experts come back to the point that assessing OA from different perspectives may be too costly or infeasible. Some also question the need.*
- *Some experts think combining structural and clinical parameters in classification systems may be difficult as they usually are not highly correlated.*
- *One expert opposes to the use of multiple classification systems, scoring 10.*

**Response:**

The difference between the fourth and second statement lies in the fact that not all differences between the consequences of phenotypes might be clinically relevant in extent and/or nature.

**Statements round 3:**

1. A system to classify OA patients for one or more phenotypes should consist of input variables that together reflect (the likelihood of) the presence of one or more pathobiological and biopsychosocial mechanisms and their structural and/or functional consequences in these patients (*score:* mean 79, SD 17, median 80).
2. To ascertain that OA phenotype classification systems could eventually influence decision-making in clinical trials and practice, the potentially identified phenotype(s) should differ from others in terms of clinically relevant disease-driving factors and/or outcomes, e.g. disease trajectory and/or responsiveness to particular treatments or treatment in general, etc. (*score:* mean 87, SD 13, median 80)
3. Classification systems could use one or more input variables from either one or more domains (e.g., imaging, biochemical, pain, etc.) to identify a clinically relevant OA phenotype or phenotypes (*score:* mean 86, SD 12, median 80).
4. Phenotype classification systems can be developed in different contexts (e.g., research vs. daily practice) and with different goals (e.g., supporting treatment decisions vs. selection of patients for studies). Therefore, there could be multiple OA phenotype classification systems that would be used either separately or in combination, e.g., systems that relate to prognosis and systems that relate to treatment response (*score:* mean 75, SD 25, median 80).

- *Some experts would rather not include the outcomes in statement 3.*
- *Some experts think the examples relating to treatment in statement 4 are confusing and/or limit the statement.*

**Response**

- The fact that clinically relevant OA phenotypes should differ with regard to functional and structural outcomes is covered in other statements. Also, outcomes are not always available at the time of the classification. We therefore agree that outcomes should not be part of the classification system itself.

**Statements round 4 (the previous statements 3 and 6):**

2. An OA phenotype classification system is likely to consist of input variables that together reflect (the likelihood of) the presence of one or more pathobiological and pain ~~and biopsychosocial~~ mechanisms ~~and their structural and/or functional consequences in these patients~~.

3. Research efforts may initially lead to multiple proposed phenotype classification systems. Eventually, these should be aligned and come together in one.

**The previous statements 4 and 5 are closed.** The wording is changed to:

- The potentially identified phenotype(s) should differ from others in terms of clinically relevant disease-driving factors and/or outcomes. (shortened)

- Classification systems are likely to use one or more measures from either one or more domains (e.g., imaging markers, biochemical markers, pain, etc.) to identify a clinically relevant OA phenotype or phenotypes.

**Statements round 1:**

- Structural OA stages (e.g., Kellgren and Lawrence grade 1 vs. 3) represent different phenotypes (*score:* mean 35, SD 25, median 30)
- Clinical OA stages (e.g., recent onset vs. long-standing pain) represent different phenotypes (*score:* mean 50, SD 30, median 50).
- OA populations for investigating phenotypes should consist of people in a similar OA stage (either clinical or structural) or analyses should at least be adjusted for that (*score:* mean 60, SD 23, median 65).
- *Most experts agree that disease stages are not similar to phenotypes.*
- *Many experts think disease stage is relevant for phenotype classification, but they differ in their argumentation.*

**Statements round 2:**

7. Disease stage is likely to be relevant for OA phenotyping, as disease stages in themselves might already differ in the predominance of etiological pathways and our ability to capture their typical characteristics (e.g. minimal vs. extensive cartilage changes, preclinical metabolic derangements vs. overt structural disease, etc.) (*score:* mean 66, SD 22, median 70).

8. It should, however, also be acknowledged that the nature and course of disease stages may differ between patients and phenotypes and that our methods for disease staging have their limitations. Reasons to adjust or not adjust analyses for disease stage should be weighed for every study (*score:* mean 80, SD 16, median 80).

- *Some authors seem to understand that the statements say that disease stages are phenotypes, like one did in the first round.*
- *One expert opposes to the word “relevant”.*
- *One expert adds that one could also look for interaction with rather than adjusting for disease stage.*
- *Some experts think these statements had better be combined or that one may replace the other.*
- *Some experts say it’s hard to define disease stages.*

**Responses:**

- We did not mean to say disease stages are similar to phenotypes. We aimed to say that disease stage might affect the results from phenotype research, as they themselves probably differ in nature and extent of pathobiological and biopsychosocial mechanisms.
- The eighth statement confirms that defining disease stage is not straightforward.

**Statements round 3:**

7. Differences in disease stage may affect the results from OA phenotyping studies, as disease stages in themselves might already differ in the predominance of etiological pathways and our ability to capture their typical characteristics (e.g. minimal vs. extensive cartilage changes, preclinical metabolic derangements vs. overt structural disease, etc.) (*score:* mean 78, SD 18, median 80).

8. It should, however, also be acknowledged that the nature and course of disease stages may differ between patients and phenotypes and that our methods for disease staging have their limitations. Reasons to take or not take disease stage into account in the analyses (e.g., to adjust for confounding or look for interaction) should be weighed for every study (*score:* mean 84, SD 13, median 80).

- *Some experts suggest combining statements 7 and 8.*
- *One expert doesn’t understand the stated differences in etiological pathways between disease stages.*

**Responses**

- Statements were numbered 4a and 4b.

- Disease stages may differ in etiological pathways, in that, for example, synovitis may be more relevant in early disease and bone metabolism in late disease. Results of a data-driven statistical analysis will therefore probably differ between early vs. late disease. Likewise, in a mixed study population of early and late disease, results might reflect differences between disease stages as well as phenotypes.

**Statements round 4 (the previous statements 7 and 8):**

4a. Differences in disease stage may cause different results from OA phenotyping studies between study populations. (simplified)

4b. Disease stage(s) of the study population should always be reported. Reasons to take or not take disease stage into account in the analyses (e.g., to adjust for confounding or look for interaction) should be weighed for every study. (simplified)

**Statement round 1:**

OA in different joints represents different phenotypes (*score:* mean 70, SD 17, median 70).

- *Most experts agree that functional or pathobiological mechanisms may to a certain extent be similar between joints, but that joint-specific mechanisms will play an important role too, e.g. menisci in the knee and femoral head shape in the hip. Moreover, some mechanisms may affect single joints, e.g., trauma, while others will affect multiple joints, e.g. metabolic factors.*
- *Multiple experts mention that phenotype classification systems can be set up either per joint type or systemically, for multiple joints in one patient, depending on the context.*

**Statements round 2**

10. Phenotype classification systems for individual joints can probably not be translated directly to other joints. Although different joints may share common functional or pathobiological OA mechanisms, their consequences probably differ between joints and joint-specific factors (e.g. menisci, femoral head shape) also play a role (*score:* mean 72, SD 19, median 75).

11. Phenotype classification systems can be set up either per joint type or systemically, for multiple joints in one patient, depending on their intended context and goal (*score:* mean 78, SD 14, median 80).

- *Some experts say that phenotypes may or may not differ between joints, depending on the phenotype.*
- *One expert thinks phenotypes on a systemic level are of no use.*
- *One expert doesn’t understand the use of “context and goal”.*
- *These two statements had better be combined.*

**Statements round 3**

9. Some components of pathobiological and biopsychosocial OA mechanisms may be similar between different joints such as knee and hip (e.g., synovitis, central pain perception), while others may differ (e.g., menisci, femoral head shape). When extrapolating findings in one joint to another joint, it should be explained why this would be appropriate (*score:* mean 86, SD 11, median 85).

10. In line with this, phenotype classification systems can be designed for individual joints or systemically, for multiple joints in one patient, depending on the pathobiological and biopsychosocial mechanism that is under study and the goal of the study (e.g., study OA impact in an individual versus predict cartilage loss in a knee) (*score:* mean 86, SD 8, median 90).

**The previous statements 9 and 10 were closed.** Statements will be numbered -a and -b:

-a. Some components of pathobiological and pain mechanisms in OA may be similar between different joints such as knee and hip (e.g., synovitis, central pain perception), while others may differ (e.g., menisci, femoral head shape). The decision to extrapolate findings from one joint to another, or not, should be justified.

-b. Phenotype classification systems can be designed for individual joints or systemically, for multiple joints in one patient, depending on the pathobiological and pain mechanism that is under study and the goal of the study. (shortened)

The underlined words will only be maintained if statement 1 is approved by the expert committee.

**Statement round 1:**

OA phenotypes derived from data-driven approaches should be valued more highly than those based on expert opinion (*score:* mean 75, SD 19, median 80).

- *Most experts tend to agree with the statement, but multiple experts emphasize the need for high-quality data and statistics for such approaches and the importance for clinical validity, relevance and applicability.*

**Statement round 2:**

12. Data-driven approaches for constructing phenotype classification systems are generally preferable over expert opinion-based approaches, as long as they are performed using high-quality data and statistics and with attention to clinical validity, relevance and applicability (*score:* mean 84, SD 15, median 80).

- *One expert suggests adding “reproducibility”.*
- *One expert suggests adding “expert opinion”.*

**Statement round 3**

1. Data-driven approaches for constructing phenotype classification systems are generally preferable over expert opinion-based approaches, as long as they are performed using high-quality data and appropriate statistics, are reproducible, and have clinical validity, relevance and applicability as judged by experts in the field (*score:* mean 91, SD 8, median 80).

**This statement is closed:** Data-driven approaches for constructing phenotype classification systems are generally preferable over expert opinion-based approaches, as long as they are performed using high-quality data and appropriate statistics, are reproducible and have clinical validity, relevance and applicability as judged by experts in the field.

**General remark:**

- *Some experts repeat that “endotypes” had better be “phenotypes”.*

**Extra statement round 4 (one-time)**

5. “Phenotypes” as they are now defined had better be replaced in all statements by “endotypes”.

**Results Delphi round 3**

| **Question 1** | OA phenotypes are subtypes of OA that share, or can reasonably be assumed to share, distinct underlying pathobiological mechanisms and their structural and functional consequences. |
| --- | --- |
| **Mean** | 82 |
| **SD** | 12 |
| **Median** | 80 |

Expert's answer: 75

Expert's comment: I would still prefer the term endotype when referring to a phenotype with a shared mechanism.

Expert's answer: 80

Expert's comment: I am not entirely convinced by the word 'pathobiological'. It takes everything into the biomedical sphere, which I'm not sure is helpful for some phenotypes, e.g. the chronic pain phenotype which can be partially explained by pathobiology (e.g. central sensitisation) but requires a psychological perspective as well.

Expert's answer: 85

Expert's comment: Simplify wording - 'OA phenotypes are subtypes of OA that can reasonably be assumed to share distinct underlying pathobiological mechanisms with potentially relevant structural and functional consequences'?

Expert's answer: 50

Expert's comment: I accept that I may be an outlier here but my hesitancies about this definition haven't changed since round 2. That is because the definition seems to be anchored in notion that the pathobiological mechanisms are exclusively central to phenotyping and that the structural and functional sequelae can only be a consequence of those mechanisms. I recognize that life would be simpler if the clinical presentation of OA was only about biology. However, while this is a traditional biomedical perspective, it is limited as it doesn't tell the whole story. Patients don't present because of OA, they present due to pain and activity limitation associated with OA and, in addition to the pathobiology, these are influenced by cognitions and emotions about what pain and OA mean to the patient. These cognitions and emotions are modifiable but vary across patient subgroups. Therefore, a definition of OA phenotypes that does not include the biopsychosocial seems incomplete.

Expert's answer: 90

Expert's comment: Improvement on Round 2 with clearer wording allowing more flexibility to include clinical presentations.

Expert's answer: 90

Expert's comment: I agree. OA consists of several (distinct) underlying mechanisms with different consequences.

Expert's answer: 70

Expert's comment: I would prefer ‘underlying pathobiological and/or biopsychosocial mechanisms and/or their structural and/or functional consequences’, as phenotypes could have similar pathogeneses but still have different trajectories or characteristics. Furthermore, the biopsychological mechanisms have been added in question 3 and should also be added here.

| **Question 2** | OA phenotypes can become apparent in differences in risk factors, prognostic factors, nature and extent of symptoms and signs, disease trajectory, and/or responsiveness to particular treatments or treatment in general. |
| --- | --- |
| **Mean** | 89 |
| **SD** | 9 |
| **Median** | 80 |

Expert's answer: 90

Expert's comment: This definition covers all the bases nicely.

Expert's answer: 90

Expert's comment: I like the use of the term endotype—it is appropriate here; since here you are trying to advance the field, I do not find the argument related to maintaining the status quo adequate justification for retaining the term phenotype

Expert's answer: 90

Expert's comment: There are several risk factors etc that the answer to this statement is that it will result in different phenotypes. It’s a very broad statement.

Expert's answer: 80

Expert's comment: Yes they can, but not necessarily

| **Question 3** | A system to classify OA patients for one or more phenotypes should consist of input variables that together reflect (the likelihood of) the presence of one or more pathobiological and biopsychosocial mechanisms and their structural and/or functional consequences in these patients. |
| --- | --- |
| **Mean** | 79 |
| **SD** | 17 |
| **Median** | 80 |

Expert's answer: 60

Expert's comment: Functional consequences of disease are an outcome, and are not necessarily known at the time one is trying to classify an individual. Requiring this may bias the classification system to those with well-established disease.

Expert's answer: 60

Expert's comment: I am not sure if variables related to outcomes need to be part of a classification system - the implications for outcomes can be tested in a second step.

Expert's answer: 95

Expert's comment: In this definition 'biopsychosocial' is included, which should also be done for the definition in Q1.

Expert's answer: 100

Expert's comment: Perfect

Expert's answer: 85

Expert's comment: The 'functional consequences' component relates more closely to the clinical presentation.

Expert's answer: 40

Expert's comment: It may be dangerous to mix pathobiological and more objective measures such as functional assessments. The the main reason for classifying patients is to provide appropiate treatment. Treatment are targeted molecular processes that may or may not provide symptomatic benefit. Thus if biopsychosocial mechanism or functional assessement is a bearing force in the classification, there is a great chance that we do not treat the disease but rather the symptoms.

Expert's answer: 40

Expert's comment: I am not sure I agree with the use of the term biopsychosocial here; if for instance pain is due to a psychological cause, this is not a subphenotype of OA, it is pain due to a different etiology; I prefer limiting the description to pathobiological

Expert's answer: 80

Expert's comment: I think the word “classify” unnecessarily confuses the issue and the statmetn is quite dense. I would delete A “system to classify OA patients for one or more”

Expert's answer: 90

Expert's comment: I agree that a system should consist input from variables reflecting these mechanisms.

Expert's answer: 70

Expert's comment: Similar as question 1: I would prefer “pathobiological and/or biopsychosocial mechanisms and/or their structural and/or functional consequences in these patients.

| **Question 4** | To ascertain that OA phenotype classification systems could eventually influence decision-making in clinical trials and practice, the potentially identified phenotype(s) should differ from others in terms of clinically relevant disease-driving factors and/or outcomes, e.g. disease trajectory and/or responsiveness to particular treatments or treatment in general, etc. |
| --- | --- |
| **Mean** | 87 |
| **SD** | 13 |
| **Median** | 80 |

Expert's answer: 75

Expert's comment: Suggest leaving it at: To ascertain that OA phenotype classification systems could eventually influence decision-making in clinical trials and practice, the potentially identified phenotype(s) should differ from others in terms of clinically relevant disease-driving factors and/or outcomes. There are a variety of outcomes that could be considered, and I am concerned that by listing just a random one or two, particularly focused on treatments (which are so limited in OA) may reduce applicability of this statement or unnecessarily limit broader thinking.

Expert's answer: 80

Expert's comment: This may not be proven necessarily upfront simply from a lack of available relevant treatments.

Expert's answer: 90

Expert's comment: Yes - I read this as: the phenotype classification needs to be clinically relevant, which I completely agree with.

Expert's answer: 50

Expert's comment: Not sure about meaning of 'clinically relevant disease-driving factors' and 'treatment in general'? The issue here seems to be about distinguishing between (phenotype) classification systems that are 'practice-relevant' vs practice-irrelevant (and patient-relevant vs patient-irrelevant). I think it is important to have a statement on this matter to encourage the pursuit of classification systems that may influence practice and patient outcomes but I think it is difficult to be prescriptive about this since it may just reflect the limits of knowledge at that time. Isn't it possible that phenotypes are discovered based on evidence of distinct underlying mechanism(s) but patients with that phenotype do not appear to differ at broader structural or symptom course under current treatment regimes that do not adequately address the underlying mechanisms?

Expert's answer: 100

Expert's comment: Absolutely

Expert's answer: 70

Expert's comment: i am not convinced by the wording of the sentence with "clinically relevant": what is a clinically relevant disease-driving factor or a clinically relevant outcome ? the differences between groups should be clinically relevant, this is not exactly what is in the sentence. "should differ from others in terms of disease-driving factors and/or outcomes, e.g. disease trajectory and/or responsiveness to particular treatments or treatment in general, etc.". The differences should be clinically relevant..."

Expert's answer: 90

Expert's comment: Phenotype classification should (in the end) influence decision-making processes. The difference is determined by particularly outcomes of interventions.

Expert's answer: 70

Expert's comment: Yes in theory

| **Question 5** | Classification systems could use one or more input variables from either one or more domains (e.g., imaging, biochemical, pain, etc.) to identify a clinically relevant OA phenotype or phenotypes. |
| --- | --- |
| **Mean** | 86 |
| **SD** | 12 |
| **Median** | 80 |

Expert's answer: 90

Expert's comment: Suggest adding the word "markers" after imaging and biochemical

Expert's answer: 95

Expert's comment: Not could - should.

Expert's answer: 80

Expert's comment: Not sure about 'input variables' - indicators, measures, biomarkers

Expert's answer: 90

Expert's comment: This definition is flexible enough to cover a number of scenarios.

Expert's answer: 70

Expert's comment: Yes as long as the output of that system is a profile and not just one number.

Expert's answer: 60

Expert's comment: Classification systems are likely to use input variables more than one domains (e.g., imaging, biochemical, pain, etc.) to identify a clinically relevant OA phenotype or phenotypes. 60 as is, 95 as above.

Expert's answer: 60

Expert's comment: Partly agreed. In my opinion a classification system should use more input variables to identify clinically phenotypes.

| **Question 6** | Phenotype classification systems can be developed in different contexts (e.g., research vs. daily practice) and with different goals (e.g., supporting treatment decisions vs. selection of patients for studies). Therefore, there could be multiple OA phenotype classification systems that would be used either separately or in combination, e.g., systems that relate to prognosis and systems that relate to treatment response. |
| --- | --- |
| **Mean** | 75 |
| **SD** | 25 |
| **Median** | 80 |

Expert's answer: 80

Expert's comment: Agree in principle but would argue for simplified wording and not having 3 e.g. abbreviations in the statement, would just leave these out: Phenotype classification systems can be developed in different contexts and with different goals. Therefore, there could be multiple OA phenotype classification systems that would be used either separately or in combination.

Expert's answer: 10

Expert's comment: I do not like the idea of multiple phenotype systems - this will lead to confusion e.g. what system is being used, why, what does the "research" phenotype mean clinically? etc etc I would MUCH rather have a single phenotype system that is applicable in all situations. If its worth phenotyping OA then we should all use the same terms all the time. You could add "descriptors" to phenotypes e.g. "rapidly progressive" inflammatory or something. BUT once the pathobiological reason for the difference is better understood - these would become distinct phenotypes.

Expert's answer: 40

Expert's comment: To be useful, systems should be aligned; enrolling study subjects should align with treatment decisions. Separate classifiation systems will not be practical.

Expert's answer: 60

Expert's comment: I think this is more classification of subgroups rather than phenotypes.

Expert's answer: 20

Expert's comment: I'm sorry, but I very much disagree. I think this will lead to confusion, and low uptake of the classification by our stakeholders. Is there one successful example from other areas of medicine where disease subtypes are classified differently depending on whether it's for research or clinical purposes?

Expert's answer: 70

Expert's comment: OK, but needs to make sense alongside question 4

Expert's answer: 90

Expert's comment: Yes, I think this the only way that it can work.

Expert's answer: 80

Expert's comment: This is complicated but reflects the complexity of the disease.

Expert's answer: 90

Expert's comment: It must be clear what the aim is of determining phenotypes.

| **Question 7** | Differences in disease stage may affect the results from OA phenotyping studies, as disease stages in themselves might already differ in the predominance of etiological pathways and our ability to capture their typical characteristics (e.g. minimal vs. extensive cartilage changes, preclinical metabolic derangements vs. overt structural disease, etc.). |
| --- | --- |
| **Mean** | 78 |
| **SD** | 18 |
| **Median** | 80 |

Expert's answer: 80

Expert's comment: Agree, but does this need to be more actionable, to say that, since differences in disease stage may affect the results, the disease stage should be characterized and stated in a given study, or something?

Expert's answer: 80

Expert's comment: I think this is part pf the preceding question - and so some of the examples given in the statement would be "descriptors" of a given phenotype e.g. "early" "progressive", "late" .... Ultimately it would be hoped that these could become unique phenotypes described e.g. by a biomarker.

Expert's answer: 70

Expert's comment: Late-stage disease would not be ideal for establishing criteria.

Expert's answer: 75

Expert's comment: That is probably true - but each phenotype should have their own characteristic course which should eventually allow us to identify which stages go with which phenotypes, rather than which phenotypes can be identified at which stages.

Expert's answer: 70

Expert's comment: Not sure what is meant by 'results from OA phenotyping studies'

Expert's answer: 90

Expert's comment: Yes, I think this nicely captures that stage may affect the results of phenotyping studies but the stages are not phenotypes.

Expert's answer: 80

Expert's comment: Could be merged with number 8.

Expert's answer: 10

Expert's comment: “disease stages in themselves might already differ in the predominance of etiological pathways”—this statement still is not understandable to me; can you provide concrete examples?

Expert's answer: 100

Expert's comment: Suggestion to combine 7 and 8: Differences in disease stage may affect the results from OA phenotyping, as disease stages in themselves might already differ in the predominance of etiological pathways and our ability to capture their typical characteristics (e.g. minimal vs. extensive cartilage changes, preclinical metabolic derangements vs. overt structural disease, etc.). It is likely that the nature and course of disease stages may differ between patients and phenotypes

Expert's answer: 80

Expert's comment: Problem with phenotyping is that the continuum of the disease process is not always covered by the defined phenotype, because variables change over time. This indicates that patients may change from phenotype over time. Question is whether it is possible to define phenotypes by longitudinal studies or should be defined only by longitudinal studies.

Expert's answer: 80

Expert's comment: This statement does not have added value, compared to the others.

| **Question 8** | It should, however, also be acknowledged that the nature and course of disease stages may differ between patients and phenotypes and that our methods for disease staging have their limitations. Reasons to take or not take disease stage into account in the analyses (e.g., to adjust for confounding or look for interaction) should be weighed for every study. |
| --- | --- |
| **Mean** | 84 |
| **SD** | 13 |
| **Median** | 80 |

Expert's answer: 80

Expert's comment: I guess this gets at my comment for Question 7, so perhaps these should be statements 7a and 7b so it is more clear?

Expert's answer: 70

Expert's comment: Not sure that i like the idea of being able to decide on when t use and not use phenotypes. If they are worth having in that they define distinct clinically relevant disease types (i.e. that have different progression, prognosis and treatment targets) then we should use the phenotypes. So if stage is worth having as a descriptor or a phenotype then it becomes its own distinct phenotype. I recognize that a study may be designed to actually better define appropriate phenotypes - e.g. only early inflammatory OA responds to bisphosphonate treatment.

Expert's answer: 75

Expert's comment: I would recommend that simply including disease duration or duration of symptoms in any models as a confounder / covariate is always done. More extensive inclusion of variables related primarily to disease stage rather than phenotype should indeed be judged individually for each study.

Expert's answer: 90

Expert's comment: Yes, this version seems to meet most of the hesitancies raised by other experts.

Expert's answer: 85

Expert's comment: Agree.

Expert's answer: 90

Expert's comment: Absolutely, which is in accordance with my previous comment.

| **Question 9** | Some components of pathobiological and biopsychosocial OA mechanisms may be similar between different joints such as knee and hip (e.g., synovitis, central pain perception), while others may differ (e.g., menisci, femoral head shape). When extrapolating findings in one joint to another joint, it should be explained why this would be appropriate. |
| --- | --- |
| **Mean** | 86 |
| **SD** | 11 |
| **Median** | 85 |

Expert's answer: 80

Expert's comment: For the second sentence, perhaps: The decision to extrapolate findings from one joint to another, or not, should be justified.

Expert's answer: 50

Expert's comment: I'm not sure without data one should extrapolate between joints. So a phenotype should probably include the joint in its name until proven that this is not necessary e.g. early inflammatory knee OA versus early inflammatory DIP OA

Expert's answer: 95

Expert's comment: Nothing to add, agree completely.

Expert's answer: 90

Expert's comment: Agree.

Expert's answer: 80

Expert's comment: I still dislike the term biopsychosocial; I think pathobiological is sufficient

Expert's answer: 90

Expert's comment: Phenotyping per joint cannot directly be used for another joint. Question is whether phenotyping should be calculated for joints separately, or on the combination of clinical characteristics.

| **Question 10** | In line with this, phenotype classification systems can be designed for individual joints or systemically, for multiple joints in one patient, depending on the pathobiological and biopsychosocial mechanism that is under study and the goal of the study (e.g., study OA impact in an individual versus predict cartilage loss in a knee). |
| --- | --- |
| **Mean** | 86 |
| **SD** | 8 |
| **Median** | 90 |

Expert's answer: 80

Expert's comment: Yes - I do think we should be able to define a succinct group of 'systemic' phenotypes which would be relevant to OA in any joint (in particular - chronic pain and inflammatory phenotypes)

Expert's answer: 90

Expert's comment: More complete with example.

Expert's answer: 80

Expert's comment: I still dislike the term biopsychosocial; I think pathobiological is sufficient

Expert's answer: 90

Expert's comment: Phenotyping for each joint can be used for scientific reasons such as finding causal factors. However, for clinical purposes the question is whether the classification of phenotypes should be calculated on more clinical characteristics (more systemically characteristics).

| **Question 11** | Data-driven approaches for constructing phenotype classification systems are generally preferable over expert opinion-based approaches, as long as they are performed using high-quality data and appropriate statistics, are reproducible, and have clinical validity, relevance and applicability as judged by experts in the field. |
| --- | --- |
| **Mean** | 91 |
| **SD** | 8 |
| **Median** | 80 |

Expert's answer: 95

Expert's comment: Yes - in particular the REPRODUCIBLE part. Science in general isn't good at confirming previous results, and I think we should be looking to reproduce findings from, for instance, the OAI cohort in other cohorts such as CHECK and MOST.

Expert's answer: 100

Expert's comment: Perfect.

Expert's answer: 90

Expert's comment: The addition of expert judgement makes this more comprehensive. It allows flexibility for the clinical applicability of modeling to be sensibly evaluated.

Expert's answer: 90

Expert's comment: The trick here is knowing the clinical data and validate, validate, validate

Expert's answer: 90

Expert's comment: Absolutely in agreement with this statement.

Expert's answer: 80

Expert's comment: Yes, not only high quality data but also enough quantity (in patient numbers and variables)
